# Safety and immunogenicity of a multivalent HIV vaccine comprising envelope protein with either DNA or NYVAC vectors (HVTN 096): a phase 1b, double-blind, placebo-controlled trial

**DOI:** 10.1016/S2352-3018(19)30262-0

**Published:** 2019-10-07

**Authors:** Giuseppe Pantaleo, Holly Janes, Shelly Karuna, Shannon Grant, G Laissa Ouedraogo, Mary Allen, Georgia D Tomaras, Nicole Frahm, David C Montefiori, Guido Ferrari, Song Ding, Carter Lee, Merlin L Robb, Mariano Esteban, Ralf Wagner, Pierre-Alexandre Bart, Nils Rettby, M Juliana McElrath, Peter B Gilbert, James G Kublin, Lawrence Corey, NIAID HIV Vaccine Trials Network

**Affiliations:** a Service of Immunology and Allergy, and Swiss Vaccine Research Institute, Lausanne University Hospital, Lausanne, Switzerland; b Vaccine and Infectious Disease Division, Fred Hutchinson Cancer Research Center, Seattle, WA, USA; c Division of AIDS, National Institute of Allergy and Infectious Diseases, National Institutes of Health, Bethesda, MD, USA; d Department of Surgery, Duke Human Vaccine Institute, Duke University Medical Center, Durham, NC, USA; e EuroVacc Foundation, Lausanne, Switzerland; f Global Solutions for Infectious Diseases, South San Francisco, CA, USA; g US Military HIV Research Program, Walter Reed Army Institute of Research, Silver Spring, MD, USA; h Henry M Jackson Foundation for the Advancement of Military Medicine, Bethesda, MD, USA; i Department of Molecular and Cellular Biology, Centro Nacional de Biotecnología, Consejo Superior de Investigaciones Científicas, Madrid, Spain; j Institute of Medical Microbiology and Hygiene, University of Regensburg, Regensburg, Germany; k Institute of Clinical Microbiology and Hygiene, University Hospital Regensburg, Regensburg, Germany; l US Centers for Disease Control and Prevention, Atlanta, GA, USA; m Bill & Melinda Gates Medical Research Institute, Cambridge, MA, USA

## Abstract

**Background:**

Up to now, immunisation regimens that have been assessed for development of HIV vaccines have included purified envelope (Env) protein among the boosting components of the regimen. We postulated that co-administration of Env protein with either a DNA or NYVAC vector during priming would result in early generation of antibody responses to the Env V1/V2 region, which are important markers for effective protection against infection. We aimed to assess the safety and immunogenicity of a multivalent HIV vaccine including either DNA or NYVAC vectors alone or in combination with Env glycoprotein (gp120) followed by a co-delivered NYVAC and Env protein boost.

**Methods:**

We did a single-centre, double-blind, placebo-controlled phase 1b trial at the Centre Hospitalier Universitaire Vaudois (Lausanne, Switzerland). We included healthy volunteers aged 18–50 years who were at low risk of HIV infection. We randomly allocated participants using computer-generated random numbers to one of four vaccination schedules or placebo (4:1), and within these schedules participants were allocated either active treatment (T1, T2, T3, and T4) or placebo (C1, C2, C3, and C4). T1 consisted of two doses of NYVAC vector followed by two doses of NYVAC vector and gp120 Env protein; T2 comprised four doses of NYVAC vector and gp120 Env protein; T3 was two doses of DNA vector followed by two doses of NYVAC vector and gp120 Env protein; and T4 was two doses of DNA vector and gp120 Env protein followed by two doses of NYVAC vector and gp120 Env protein. Placebo injections were matched to the corresponding active treatment group. Doses were administered by injection at months 0, 1, 3, and 6. Primary outcomes were safety and immunogenicity of the vaccine schedules. Immune response measures included cross-clade and epitope-specific binding antibodies, neutralising antibodies, and antibody-dependent cell-mediated cytotoxicity measured 2 weeks after the month 1, 3, and 6 vaccinations. This trial is registered with ClinicalTrials.gov, NCT01799954.

**Findings:**

Between Aug 23, 2012, and April 18, 2013, 148 healthy adult volunteers were screened for the trial, of whom 96 participants were enrolled. 20 individuals were allocated to each active treatment group (groups T1–4; n=80) and four were assigned to each placebo group (groups C1–4; n=16). Vaccines containing the NYVAC vector (groups T1 and T2) were associated with more frequent severe reactogenicity and more adverse events than were vaccines containing the DNA vector (groups T3 and T4). The most frequent adverse events judged related to study product were lymphadenopathy (n=9) and hypoaesthesia (n=2). Two participants, one in the placebo group and one in the DNA-primed T3 group, had serious adverse events that were judged unrelated to study product. One participant in the T3 group died from cranial trauma after a motor vehicle accident. Across the active treatment groups, IgG responses 2 weeks after the 6-month dose of vaccine were 74–95%. Early administration of gp120 Env protein (groups T2 and T4) was associated with a substantially earlier and higher area under the curve for gp120 Env binding, production of anti-V1/V2 and neutralising antibodies, and better antibody-response coverage over a period of 18 months, compared with vaccination regimens that delayed administration of gp120 Env protein until the 3-month vaccination (groups T1 and T3).

**Interpretation:**

Co-administration of gp120 Env protein components with DNA or NYVAC vectors during priming led to early and potent induction of Env V1/V2 IgG binding antibody responses. This immunisation approach should be considered for induction of preventive antibodies in future HIV vaccine efficacy trials.

**Funding:**

National Institutes of Health, National Institute of Allergy and Infectious Diseases, and the Bill & Melinda Gates Foundation.

**Research in context**

**Evidence before this study**

We searched PubMed between 2005 and 2012 for preclinical and clinical studies of HIV vaccination schedules incorporating co-administration of DNA vector in combination with envelope (Env) proteins during priming and boosting phases. Several preclinical studies have shown promising results of such a vaccination schedule conferring protection from infection; however, similar schedules have not been tested in clinical trials.

**Added value of this study**

We did a double-blind, placebo-controlled, phase 1b, clinical trial in healthy adult volunteers at low risk of HIV infection. Participants were allocated to one of four multicomponent HIV vaccine schedules that included priming with either DNA or NYVAC vectors alone or in combination with Env glycoprotein (gp120) followed by a co-delivered NYVAC and Env protein boost. Vaccines containing the NYVAC vector were associated with more frequent severe reactogenicity and more adverse events than were vaccines containing the DNA vector. Immune response measures included cross-clade and epitope-specific binding antibodies, neutralising antibodies, and antibody dependent cell-mediated cytotoxicity. IgG antibody responses were high after vaccination across all active treatment groups. Early administration of gp120 Env protein (ie, during priming) was associated with a substantially earlier and higher induction of gp120 Env binding and antibodies against the V1/V2 region and with better antibody response coverage over a period of 18 months compared with regimens delaying gp120 Env protein administration until the 3-month vaccination.

**Implications of all the available evidence**

DNA vectors in combination with Env proteins provide vaccination regimens capable of inducing potent antibody responses. These responses were identified as immune correlates of protection in the RV144 trial. Therefore, a DNA vector and Env protein vaccine could represent a valuable simplification in HIV vaccination regimens aimed at inducing antibody responses. Further research in clinical efficacy trials is needed to correlate the value of the rapid appearance of antibody responses with protection.

## Introduction

HIV vaccine development over the past 35 years has tested several different ideas for vaccines and vaccination regimens. The primary mechanism of protection of most licensed vaccines is with antibodies. Early ideas that were tested included HIV envelope (Env) protein-based vaccines with the objective of inducing neutralising antibodies. In two efficacy trials, called VAX003 and VAX004,^1^, ^2^ an Env glycoprotein subunit-based vaccine (recombinant monomeric gp120) was tested in two populations at risk of HIV infection in Thailand. In these two trials, bivalent monomeric gp120 induced type-specific, but not heterologous, neutralising antibodies against primary HIV-1 isolates and did not show protection against HIV infection.

The absence of induction of protective antibody responses caused a substantial strategic shift in the area of HIV vaccines. Based on the importance of HIV-specific CD8 T cells in the control of virus replication during chronic infection, interest shifted from antibody-mediated vaccines to candidates inducing HIV-specific cellular immunity. Vaccine candidates included viral vectors such as poxvirus and adenovirus and DNA vectors expressing predominantly HIV structural proteins. An adenovirus type 5 (Ad5) vector-based vaccine expressing the Gag, Pol, and Nef proteins was tested in two vaccine trials, Step^3^ and Phambili.^4^ This Ad5 vector-based vaccine showed no protection against HIV, and increased risk of HIV infection was seen in some vaccinated subgroups versus placebo in the Step trial.^5^, ^6^, ^7^ In another trial,^8^ a vaccine regimen consisting of a DNA vector prime and a different Ad5 vector boost did not protect against HIV infection. Both the DNA and Ad5 vector-based vaccines expressed A, B, and C gp140 Env proteins in addition to the Gag and Pol proteins (Nef protein was expressed by the DNA vector-based vaccine only).

These trials of vector-based vaccines showed that multiple immunisations (in particular, heterologous prime and boost combinations with two different vectors) could induce potent and long-lasting cellular immunity. Subsequently, the area of HIV vaccine development shifted back to the antibody idea with the variation of designing viral vectors or DNA and viral vector regimens in combination with Env proteins. In the phase 3 RV144 trial,^9^, ^10^ a prime and boost regimen consisting of a recombinant canarypox vector expressing HIV-1 Env protein, Gag protein, and protease (ALVAC-vCP1521) and bivalent recombinant monomeric gp120 Env protein (AIDSVAX B/E) showed modest protection (31%) 42 months after the first vaccination despite the absence of neutralising antibodies. Binding IgG antibodies directed at conserved regions of the V1/V2 loop, and antibodies mediating antibody-dependent cellular cytotoxicity (ADCC) in the presence of low plasma IgA, correlated with reduced risk of HIV infection.^11^, ^12^ The highest level of protection from HIV infection (60%) was seen during the first year of follow-up after the first vaccination in RV144, then protection waned over time in association with a drop in anti-V1/V2 titres,^11^, ^12^ indicating that regimens eliciting high and durable levels of antibodies to HIV might have enhanced efficacy. IgG3 binding antibody responses to gp120 Env protein correlated strongly with reduced risk of HIV infection in the RV144 trial.^13^

In the HIV Vaccine Trials Network (HVTN) 096 study, we aimed to test the hypothesis that co-administration of vaccines containing Env protein during priming in a heterologous prime and boost regimen would induce early generation of an antibody response against the V1/V2 region. Previous studies in mice and macaques showed robust early antibody development if protein was co-administered during priming.^14^, ^15^ A novel immunisation schedule was designed with the objective of accelerating the induction of protective antibody responses by vaccination, thereby extending the period of protection seen in the RV144 trial. We aimed to compare vaccine regimens comprising co-administration during priming of either a DNA or NYVAC vector and gp120 Env protein versus DNA or NYVAC vectors alone followed by a NYVAC vector and gp120 protein boost.

## Methods

### Study design and participants

HVTN 096 is a single-centre, double-blind, randomised, placebo-controlled phase 1b trial done at the HVTN clinical research site at Centre Hospitalier Universitaire Vaudois (CHUV) in Lausanne, Switzerland. We included in our trial healthy adult volunteers judged to be at low risk of HIV infection. To meet inclusion criteria for enrolment, individuals had to be aged 18–50 years and in good general health, complete a questionnaire showing understanding of the study, be able and willing to provide informed consent, have access to a participating HVTN clinical research site, be willing to be followed up for the planned duration of the study and to be contacted annually after completion of scheduled clinic visits for a total of 5 years after the initial study injection, agree not to enrol in another study of an investigational research agent, be willing to receive HIV test results, be amenable to HIV risk reduction counselling, and be willing to be assessed by clinic staff for their risk for HIV infection. Pregnant women were excluded; volunteers who could become pregnant had to agree to consistently use effective contraception and to not seek pregnancy through alternative methods. Laboratory inclusion criteria, tested within 56 days before study enrolment, included negative HIV-1 and HIV-2 tests, amounts of aspartate aminotransferase, alanine aminotransferase, alkaline phosphatase, and creatinine lower than 1·1 times the institutional upper limit of normal, negative blood tests for chronic hepatitis B and C, normal urine on dipstick (ie, no glucose, no [or trace] protein, and no [or trace] haemoglobin; if trace haemoglobin was present on dipstick, red blood cells had to be within the institutional normal range on microscopic urinalysis), a haemoglobin amount greater than or equal to 11·0 g/dL for volunteers who were born female and greater than or equal to 13·0 g/dL for volunteers who were born male, a white-blood-cell count of 3300–12 000 cells per μL, a total lymphocyte count greater than or equal to 800 cells per μL, remaining differential either within the institutional normal range or with site doctor approval, and a platelet count of 125 000–550 000 cells per μL.

All study participants provided written informed consent before participation. The study protocol was approved by the institutional ethics committee of CHUV and by Swissmedic, the Swiss Agency for Therapeutic Products (Bern, Switzerland). The trial was overseen by the HVTN safety monitoring board.

### Randomisation and masking

Participants were randomly allocated to one of four vaccination schedules (table); within these schedules, participants were assigned (4:1) to either active treatment (groups T1, T2, T3, and T4) or placebo (groups C1, C2, C3, and C4). The randomisation sequence was obtained by computer-generated random numbers and provided to the HVTN clinical research site through the HVTN statistical and data management centre's web-based randomisation system. The randomisation procedure was done in blocks (sizes 20, 24, 24, and 28) to ensure balance across vaccination schedules. The pharmacist with primary responsibility for dispensing study products maintained security of the treatment assignments.

**Table:** Vaccination schedules and masking of interventions

		**Month 0**	**Month 1**	**Month 3**	**Month 6**
**Vaccination schedule 1**					
T1 (n=20)					
	Left deltoid	NYVAC vector (2 injections)	NYVAC vector (2 injections)	NYVAC vector (2 injections)	NYVAC vector (2 injections)
	Right deltoid	Placebo*	Placebo*	gp120 Env protein†	gp120 Env protein†
C1 (n=4)					
	Left deltoid	Placebo* (2 injections)	Placebo* (2 injections)	Placebo* (2 injections)	Placebo* (2 injections)
	Right deltoid	Placebo*	Placebo*	Placebo‡	Placebo‡
**Vaccination schedule 2**					
T2 (n=20)					
	Left deltoid	NYVAC vector (2 injections)	NYVAC vector (2 injections)	NYVAC vector (2 injections)	NYVAC vector (2 injections)
	Right deltoid	gp120 Env protein†	gp120 Env protein†	gp120 Env protein†	gp120 Env protein†
C2 (n=4)					
	Left deltoid	Placebo* (2 injections)	Placebo* (2 injections)	Placebo* (2 injections)	Placebo* (2 injections)
	Right deltoid	Placebo‡	Placebo‡	Placebo‡	Placebo‡
**Vaccination schedule 3**					
T3 (n=20)					
	Left deltoid	DNA vector + placebo*	DNA vector + placebo*	NYVAC vector (2 injections)	NYVAC vector (2 injections)
	Right deltoid	Placebo*	Placebo*	gp120 Env protein†	gp120 Env protein†
C3 (n=4)					
	Left deltoid	Placebo* (2 injections)	Placebo* (2 injections)	Placebo* (2 injections)	Placebo* (2 injections)
	Right deltoid	Placebo*	Placebo*	Placebo‡	Placebo‡
**Vaccination schedule 4**					
T4 (n=20)					
	Left deltoid	DNA vector + placebo*	DNA vector + placebo*	NYVAC vector (2 injections)	NYVAC vector (2 injections)
	Right deltoid	gp120 Env protein†	gp120 Env protein†	gp120 Env protein†	gp120 Env protein†
C4 (n=4)					
	Left deltoid	Placebo* (2 injections)	Placebo* (2 injections)	Placebo* (2 injections)	Placebo* (2 injections)
	Right deltoid	Placebo‡	Placebo‡	Placebo‡	Placebo‡

Placebo injections were used to equalise the number of injections among groups and achieve masking of assignments. T=treatment group. C=control group. Env=envelope.

* Sodium chloride (0·9%) was used as the placebo for NYVAC and DNA vectors.

† AIDSVAX B/E.

‡ 600 μg of aluminium hydroxide adjuvant was used as the placebo for AIDSVAX B/E.

To achieve masking of participants and treating clinicians, injections were administered at months 0, 1, 3, and 6 in all active treatment and placebo groups, and all participants received injections at every timepoint, two in the left arm and one in the right arm (table). Active treatment groups T1 and T3 were primed at months 0 and 1 with either the NYVAC (T1) or DNA (T3) vector, and groups T2 and T4 were primed at months 0 and 1 with gp120 Env protein co-administered with either NYVAC (T2) or DNA (T4) vector. All active treatment groups received the NYVAC vector and gp120 Env protein co-administration at months 3 and 6. Placebo groups (C1–4) received injections matched with the corresponding active treatment. All injections (1 mL) were administered intramuscularly in the deltoid; gp120 Env protein or placebo was administered contralaterally to either the DNA or NYVAC vector or placebo.

### Procedures

The modified NYVAC and DNA vectors used in our study have been described elsewhere.^16^, ^17^ The DNA vector (DNA-HIV-PT123; expressing HIV-1 clade C 96ZM651gp140, 96ZM651Gag, and CN54PolNef) was administered at 4 mg/mL. The recombinant NYVAC vector consisted of two injections of NYVAC-HIV-PT1 (expressing HIV-1 clade C 96ZM651gp140) and NYVAC-HIV-PT4 (expressing HIV-1 clade C 96ZM651Gag fused to HIV-1 clade C CN54PolNef)^16^ at a final dose of about 1·2 x 10^8^ plaque-forming units, with release assay variability of 0·5 log_10_. gp120 Env protein (AIDSVAX B/E; clade B MNgp120 and clade E A244gp120) was administered at 600 μg/mL (300 μg of each glycoprotein) adsorbed onto 600 μg of aluminium hydroxide gel adjuvant. Placebo groups received 600 μg of aluminium hydroxide adjuvant instead of gp120 Env protein and 0·9% sodium chloride instead of the DNA or NYVAC vector.

Samples of serum and peripheral blood mononuclear cells (PBMCs) were obtained 2 weeks after the month 1, 3, and 6 vaccinations, then 6 months after the month 6 vaccination. Serum samples were additionally obtained at baseline (month 0) and at 3, 9, and 12 months after the month 6 vaccination. The primary timepoint for assessing peak immunogenicity for all assays was 2 weeks after the month 6 vaccination. The primary durability timepoint was 12 months after the month 6 vaccination for binding antibodies and 6 months after the month 6 vaccination for neutralising antibodies and T cells.

Serum samples for humoral assays were obtained from serum-separating tubes and frozen at −80°C until use. PBMCs for cellular assays were isolated as described previously.^18^

Intracellular cytokine staining (ICS) was done on cryopreserved PBMCs by flow cytometry to examine HIV-1-specific vaccine-induced CD4 and CD8 T-cell responses at 2 weeks after the month 1, 3, and 6 vaccinations and at 6 months after the month 6 vaccination. Cytokine production was assessed at these same timepoints after stimulation with global potential T-cell epitope (PTE_g_) peptide pools^19^ representing 15-mer peptides (Gag-1-PTE_g_, Gag-2-PTE_g_, Pol-1-PTE_g_, Pol-2-PTE_g_, Pol-3-PTE_g_, Env-1-PTE_g_, Env-2-PTE_g_, Env-3-PTE_g_, and Nef-PTE_g_; Bio-Synthesis, Lewisville, TX, USA).^19^, ^20^ Phytohaemagglutinin (Remel; ThermoFisher Scientific, Lenexa, KS, USA) was used as a positive control; peptide diluent (dimethyl sulphoxide at a final concentration of 1%) was used as a negative control. Cells were stained with Live/Dead Fixable Aqua Dead Cell Stain (Life Technologies, Carlsbad, CA, USA) and intracellularly with fluorescently labelled antibodies to CD14 (exclusion marker), CD3, CD4, CD8, interferon γ, and interleukin 2.^20^ Data were acquired on an LSR II flow cytometer (BD Biosciences, San Jose, CA, USA) and analysed using FlowJo (Tree Star, Ashland, OR, USA). Positivity of ICS responses of individual cytokines or cytokine combination was ascertained by a one-sided Fisher's exact test, as described previously.^20^

Neutralising antibodies were measured as a function of reduction in luciferase (*luc*) reporter gene expression after one round of infection in TZM-bl cells.^21^ Assay stocks of molecularly cloned Env-pseudotyped viruses were prepared by transfection in 293T/17 cells (American Type Culture Collection, Manassas, VA, USA) and titrated in TZM-bl cells, as described previously.^21^ This assay has been formally optimised and validated^22^ and was done in compliance with Good Clinical Laboratory Practices. Additional information on the assay and all supporting protocols are available elsewhere.^23^ Neutralisation ID_50_ titres (ie, the number of virus particles needed to produce infection in 50% of people) 2 weeks after the month 1, 3, and 6 vaccinations and 6 months after the month 6 vaccination were measured by the HIV neutralising antibody assay. Serum samples were assayed against a panel of six heterologous Env-pseudotyped viruses that show tier 1 neutralisation, and against one of the vaccine strains (96ZM651.2, clade C, tier 2) and a global panel of nine heterologous tier 2 Env-pseudotyped viruses 2 weeks after the month 6 vaccination (appendix p 7). Magnitude-breadth curves^24^ were used to summarise responses across viruses.

Binding antibody assays were done as described previously.^11^, ^25^ The frequency and magnitude of IgG and IgA Env-specific binding antibodies were measured by the HIV-1 binding antibody multiplex assay (BAMA), from 2 weeks after the month 1 vaccination until 12 months after the month 6 vaccination. The primary positive response indicator was a positive response to any of the vaccine-matched antigens 96ZM65.1 gp140, A244 gDneg/293F/mon gp120, or MN gp120 gDneg/293F/mon gp120 (termed the aggregate vaccine-matched response). The primary magnitude of response was the geometric mean of the blank-subtracted readouts across the vaccine-matched antigens. Binding antibodies to additional antigens included group M consensus ConS gp140 CFI (ConS gp140), gp120 proteins (Con6 gp120/B [Con6 gp120], MN gp120 gDneg/293F [MN gp120]), V1/V2 proteins (AE.A244 V1V2 Tags/293F, gp70 case_B.CaseA_V1_V2 and gp70_B.CaseA2 V1/V2/169K), gp41 (clade B), and p24 Gag. Further details of antigens used are in the appendix (p 7). Use of V1/V2 antigens was based on previous findings from the RV144 study^11^, ^26^ indicating that IgG antibodies to V1/V2 regions correlated with reduced risk of HIV infection. With respect to the V1/V2/169 antigen, vaccine efficacy against viruses matching the vaccine at position 169 (Lys169) was 48%, whereas vaccine efficacy versus position 169-mismatched viruses was not significant.^27^ Therefore, responses against Lys169 were identified as a correlate of protection in the RV144 study.

ADCC-mediated antibody responses were measured by the ADCC GranToxiLux (GTL) assay and were tested against the vaccine-matched antigens 96ZM65.1_∆11gp120.avi/293F (ZM96 gp120), AE.A244_gDneg_gp120/293F (A244 gp120), and MN_gp120gDneg/293F monomer (MN gp120) using gp120-coated cells (percentage granzyme B readout). Participants' serum samples were incubated with effector cells and gp120-coated target cells^28^ and ADCC was quantified as the net percentage granzyme B activity, which is the percentage of target cells positive for GTL detected by flow cytometry. A positive response was defined as peak activity greater than or equal to 8%. The primary measure of the magnitude of response was the non-parametric area under the net percentage granzyme B activity versus log_10_ (dilution) curve (AUC). The secondary magnitude measure was peak activity.

### Outcomes

The first primary outcome was to assess the safety and tolerability of the four vaccination schedules, which we measured by recording local and systemic reactogenicity signs and symptoms, laboratory measures of safety, and adverse events and serious adverse events. The site principal investigator was initially responsible for assessing and reporting safety events, which were then reviewed weekly by a Protocol Safety Review Team (PSRT) consisting of doctors who were the Chair and co-Chair of the protocol, the funder's medical officer, and an HVTN medical monitor, and a clinical safety specialist who was a nurse. An independent safety monitoring board consisting of specialists in infectious disease, virology, immunology, and vacccinology also reviewed safety events quarterly. The safety monitoring board reviewed safety data while aware of treatment allocations, whereas the principal investigator and PSRT were unaware of treatment allocations. Details of local and systemic reactogenicity will be published in a separate report.

The other two primary outcomes were to assess immunogenicity and durability of the four vaccination schedules and to compare immunogenicity and durability between the NYVAC vector alone and NYVAC vector plus gp120 Env protein prime strategies (T1 *vs* T2) and between the DNA vector alone and DNA vector plus gp120 Env protein prime strategies (T3 *vs* T4). Immune response measures were HIV-specific cross-clade binding IgG Env antibody responses 2 weeks after the month 6 vaccination (for immunogenicity) and between 2 weeks and 12 months after the month 6 vaccination (for durability). Secondary outcomes were to assess immunogenicity of the four priming regimens at 2 weeks after the month 1 vaccination, to compare immunogenicity between priming regimens at 2 weeks after the month 1 vaccination (T1 *vs* T2 and T3 *vs* T4) and at 2 weeks after the month 3 and 6 vaccinations (T1 *vs* T2, T3 *vs* T4, T1 *vs* T3, and T2 *vs* T4), and to assess durability of vaccine-induced immune responses at 6 months after the month 6 vaccination. Immune response measures were cross-clade and epitope-specific binding antibodies, neutralising antibodies, and ADCC. Further details of study objectives, outcomes, and outcome measures are in the study protocol.

### Statistical analysis

All participants contributed to the safety analysis. Only participants with samples available and meeting assay-specific quality-control criteria were included in immunogenicity analyses. Positive responses were compared using Fisher's exact test. The magnitudes of responses among positive responders (AUC and peak activity) were compared using Wilcoxon rank sum tests. 95% CIs were calculated using the score test method.^29^ To assess the area under the IgG binding antibody response curve among fully vaccinated participants, missing binding antibody responses were imputed using predictive mean matching,^30^ separately for each antigen, with study group, sex, and previous binding antibody responses as predictors, using the R package Mice.^31^ AUC was estimated using the trapezoidal rule, with responses below 100 truncated. Log_10_-AUC values were compared between vaccine groups using the *t* test, and results were pooled across imputed datasets using standard rules.^32^ No adjustment for multiple comparisons was done for multiple immune response assays or timepoints. p values were judged significant at the 0·05 level. In view of the nature and sample size of this phase 1b trial, a somewhat increased type I error that might result from not doing formal multiplicity correction was preferable to increase power to detect differences among vaccine groups.

This trial is registered with ClinicalTrials.gov, NCT01799954.

### Role of the funding source

GLO and MA are employed by the funder. The funder contributed to, reviewed, and approved the study's design, data analysis, and preparation of the report and concurred with the decision to submit for publication. The funder had no role in data collection or statistical analyses. The corresponding author had full access to all data in the study and had final responsibility for the decision to submit for publication.

## Results

**Figure 1:**
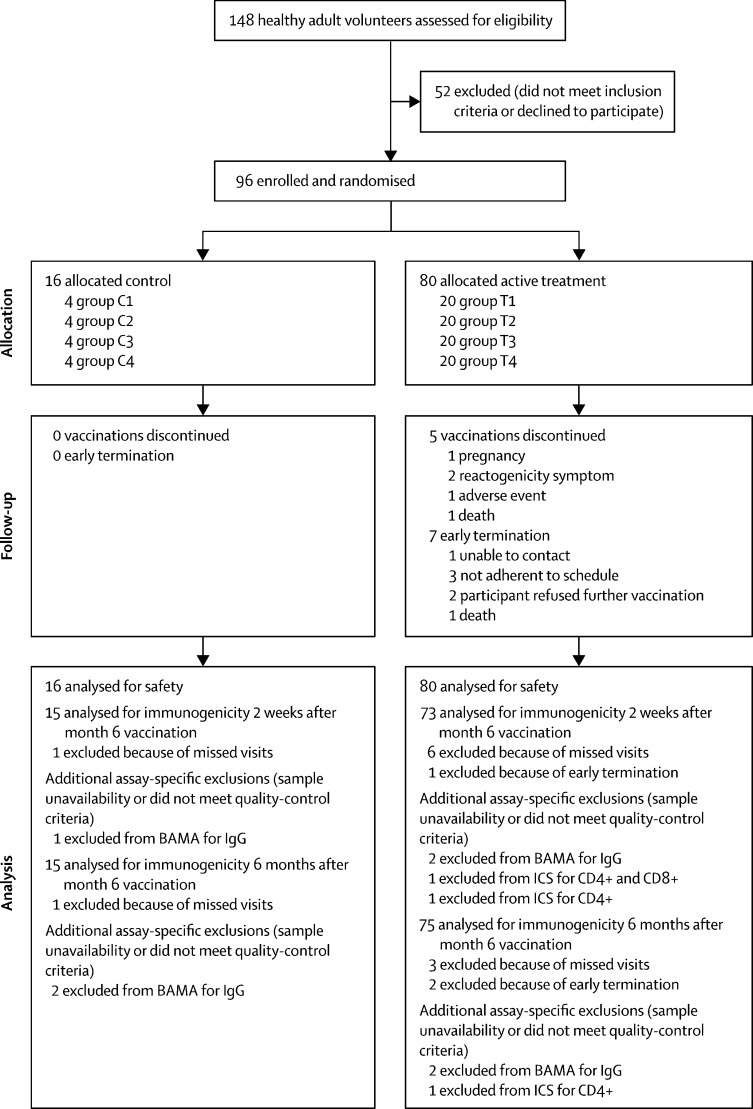
Trial profile C=control. T=treatment. BAMA=binding antibody multiplex assay. ICS=intracellular cytokine staining.

Between Aug 23, 2012, and April 18, 2013, 148 healthy adult volunteers (aged 18–50 years) were screened for trial eligibility. 96 individuals at low risk of HIV infection were enrolled, of whom 47 (49%) were male and 49 (51%) were female (appendix p 2). 60 (63%) participants were aged 21–30 years and 72 (75%) were of non-Hispanic white ethnic origin. 20 participants (figure 1) were randomly allocated to each active treatment group (T1, T2, T3, and T4) and four individuals were assigned to each placebo group (C1, C2, C3, and C4) .

Vaccines containing NYVAC vector (groups T1 and T2) were associated with more frequent severe reactogenicity and more adverse events (appendix pp 3–6) than were vaccines containing DNA vector (groups T3 and T4). 91 participants had one or more adverse events, of which most were mild (n=32) or moderate (n=51). Adverse events judged related to study product were lymphadenopathy (n=9), hypoaesthesia (n=2), injection-site pruritis (n=1), dizziness (n=1), photosensitivity (n=1), maculopapular rash (n=1), and palpitations (n=1).

Three serious adverse events were recorded: one participant who received placebo (group C1) had severe Leber's hereditary optic neuropathy; one participant in the DNA-primed T3 group had severe somatoform disorder; and one participant in the T3 group died from cranial trauma after a motor vehicle accident. No serious adverse events or deaths in this study were judged related to study product administration.

**Figure 2:**
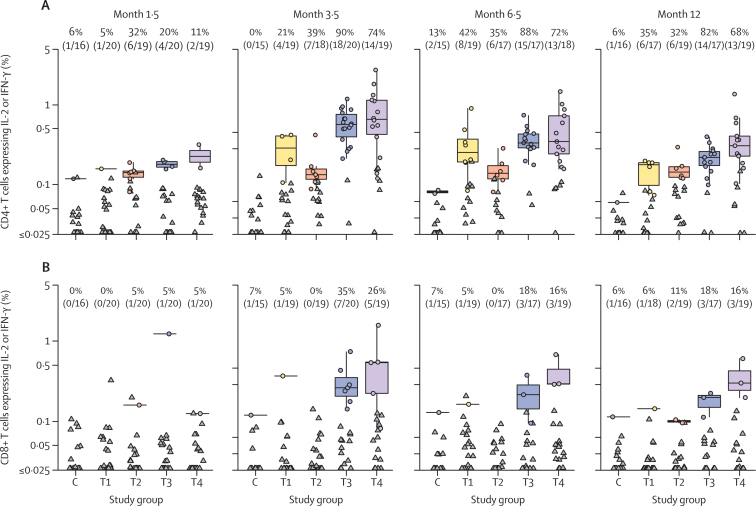
T-cell responses at different timepoints after vaccine administration CD4 (A) and CD8 (B) T-cell responses were measured by intracellular cytokine staining and expressed as the percentage of cells producing IL-2, IFN-γ, or both in the different treatment groups (T1–T4) and the control group (C). CD4 and CD8 T-cell responses and magnitudes of response among positive responders to any HIV PTE_g_ are shown 2 weeks after the month 1 (month 1·5), month 3 (month 3·5), and month 6 (month 6·5) vaccinations and 6 months after the month 6 vaccination (month 12). The proportion of positive responders for every study group is shown at the top of every panel. Horizontal lines depict median responses. Boxplots represent the distribution of responses. Circles denote positive responders. Triangles represent negative responders. T1=two doses of NYVAC vector followed by two doses of NYVAC vector and gp120 Env protein. T2=four doses of NYVAC vector and gp120 Env protein. T3=two doses of DNA vector followed by two doses of NYVAC vector and gp120 Env protein. T4=two doses of DNA vector and gp120 Env protein followed by two doses of NYVAC vector and gp120 Env protein. C=placebo groups (C1–C4), comprising matched injections. IFN=interferon. IL=interleukin. PTE_g_=global potential T-cell epitope. Env=envelope.

ICS assays showed that CD4 T-cell responses were more common than CD8 T-cell responses. The highest CD4 T-cell responses and magnitudes to any HIV PTE_g_ were noted 2 weeks after the month 3 vaccination in the DNA vector-primed groups T3 and T4, with responses seen in 90% and 74% of participants, respectively, and with median response magnitudes of 0·57% and 0·66% CD4 cells expressing interleukin 2, interferon γ, or both, respectively (figure 2A). CD4 T-cell responses for the NYVAC vector-primed groups T1 and T2 peaked at a later timepoint, 2 weeks after the month 6 vaccination, and were lower in frequency (42% and 35%, respectively) and magnitude (median 0·26% and 0·14% CD4 T cells expressing interleukin 2, interferon γ, or both, respectively) than were the peak responses in the DNA vector-primed groups. 2 weeks after the month 6 vaccination, CD4 T-cell responses were significantly higher in DNA vector-primed groups compared with NYVAC vector-primed groups (p=0·0058 for T1 *vs* T3 and p=0·044 for T2 *vs* T4). CD8 T-cell responses peaked 2 weeks after the month 3 vaccination for DNA vector-primed groups (35% in T3 group and 26% in T4 group) and were low at all timepoints in the NYVAC vector-primed groups (best response was 6 months after month 6 vaccination, 6% in T1 group and 11% in T2 group; figure 2B). CD8 T-cell responses were significantly higher in DNA vector-primed groups than in NYVAC vector-primed groups 2 weeks after the month 3 vaccination (p=0·044 for T1 *vs* T3 and p=0·046 for T2 *vs* T4). Both CD4 and CD8 T-cell responses were primarily directed to Env and Gag proteins. CD4 and CD8 T-cell responses to Gag protein had a profile similar to those to Env protein (appendix p 9).

**Figure 3:**
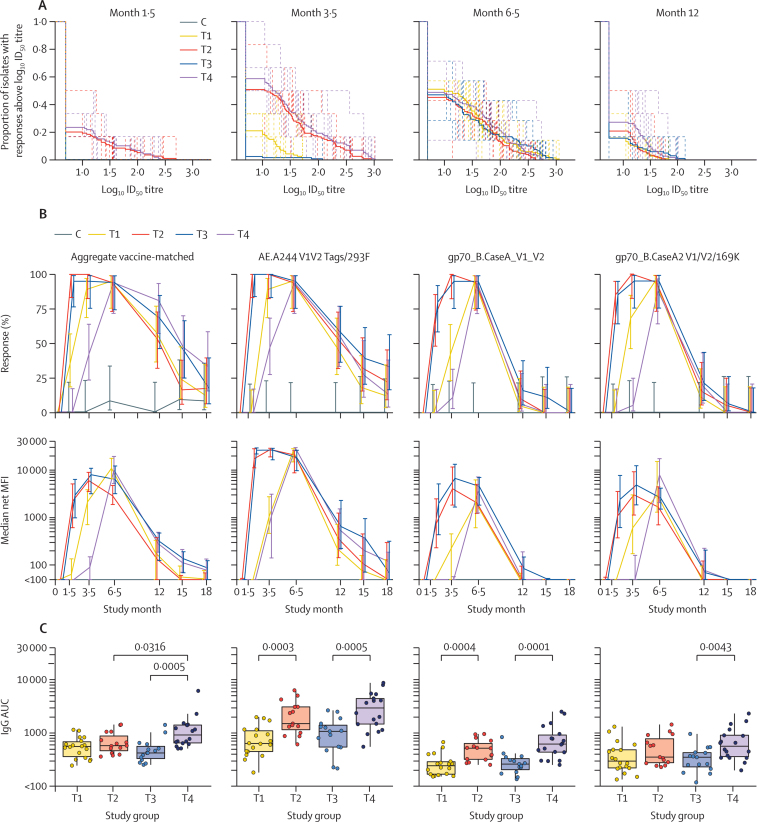
Antibody responses at different timepoints after vaccine administration (A) Neutralising antibody magnitude–breadth curves are shown against a panel of six Env-pseudotyped tier 1 HIV-1 isolates (BaL.26, MN.3, SF162.LS, MW965.26, NP03.13, and TH023.6) 2 weeks after the month 1 (month 1·5), month 3 (month 3·5), and month 6 (month 6·5) vaccinations and 6 months after the month 6 vaccination (month 12). The AUC, interpretable as the geometric mean ID_50_ titre across viruses, was used to compare groups. Individual participants are dashed lines and treatment group averages are solid lines. (B) IgG binding antibody responses and magnitudes among positive responders were measured by binding antibody multiplex assays 2 weeks after the month 1 (month 1·5), month 3 (month 3·5), and month 6 (month 6·5) vaccinations, and the durability of the antibody response was measured 6 months (month 12), 9 months (month 15), and 12 months (month 18) after the month 6 vaccination. IgG binding antibodies were measured against three vaccine-matched antigens (aggregate vaccine-matched responses) and three V1/V2 antigens. Responses to aggregate vaccine-matched antigens were judged positive if a positive response was seen to any of the vaccine-matched antigens. Errors bars show 95% CIs (upper panels) or IQRs (lower panels). (C) IgG binding magnitude AUC values are shown against aggregate vaccine-matched antigens and three V1/V2 antigens. Net MFI values are shown, and net MFI values less than 100 were truncated for MFI calculation. The AUC from months 0–18 (month 0 vaccination to 12 months after the month 6 vaccination) was calculated using the trapezoidal rule for every fully vaccinated participant. Boxplots represent the distribution of positive responders with net MFI values greater than 100. T1=two doses of NYVAC vector followed by two doses of NYVAC vector and gp120 Env protein. T2=four doses of NYVAC vector and gp120 Env protein. T3=two doses of DNA vector followed by two doses of NYVAC vector and gp120 Env protein. T4=two doses of DNA vector and gp120 Env protein followed by two doses of NYVAC vector and gp120 Env protein. C=placebo groups (C1–C4), comprising matched injections. Env=envelope. AUC=area under the curve. ID_50_=number of virus particles needed to produce infection in 50% of people. MFI=mean fluorescence intensity.

gp120 Env protein co-administration during priming (groups T2 and T4) resulted in earlier peak neutralising antibody responses than did regimens without gp120 Env protein at prime (groups T1 and T3) 2 weeks after the month 3 vaccination compared with 2 weeks after the month 6 vaccination (figure 3A). Additionally, at both 2 weeks after the month 1 vaccination and 2 weeks after the month 3 vaccination, groups with gp120 Env protein co-administration during priming (groups T2 and T4) had a significantly higher AUC than did groups without gp120 Env protein co-administration at prime (groups T1 and T3; p<0·0001 for T1 *vs* T2 and p<0·0001 for T3 *vs* T4, at both timepoints). Priming with the NYVAC vector alone (group T1) versus the DNA vector alone (group T3) yielded a higher AUC 2 weeks after the month 3 vaccination (p=0·0007 for T1 *vs* T3). Yet, the early effects of gp120 Env protein co-administration (groups T2 and T4) and of DNA vector priming (groups T3 and T4) versus NYVAC vector priming (groups T1 and T2) were no longer apparent at later timepoints. No positive responses to the vaccine-matched antigen 96ZM651.2 were detected in any vaccine group nor to any other tier 2 viruses (data not shown). Overall, the neutralising antibody responses mainly targeted tier 1 strains of HIV-1 and cross-neutralisation was seen against multiple genetic subtypes of tier 1 viruses (clades B, C and CRF01_AE; appendix p 8).

Aggregate vaccine-matched and gp140 and gp120 mismatched IgG responses were detectable at 2 weeks after the month 1 vaccination in gp120 Env protein co-administration groups during priming (groups T2 and T4), with vaccine-matched responses of 100% in T2 and 95% in T4 (figure 3B; appendix p 10). These early responses peaked 2 weeks after the month 3 vaccination and remained flat or slightly decreased 2 weeks after the month 6 vaccination. By contrast, binding antibodies were low or undetectable until 2 weeks after the month 3 vaccination in groups T1 and T3 (after administration of the first dose of gp120 Env protein) and peaked 2 weeks after the month 6 vaccination, at which time vaccine-matched responses were 95% in the T1 group and 94% in the T3 group. Binding antibody responses were infrequent and low in magnitude 12 months after the month 6 vaccination in all groups. These patterns were similar for IgG responses to the V1/V2 region of gp120 (figure 3B). IgG responses 2 weeks after the month 3 vaccination were high for all antigens (74–100% overall, 94–95% to the vaccine insert).

IgG responses were detected earlier with NYVAC vector priming only (group T1) compared with DNA vector priming only (T3), with responses 2 weeks after the month 3 vaccination of 89% versus 42%, respectively (p=0·0051). The difference between these two groups waned over time, and responses and magnitudes of response were similar after the month 6 vaccination. In groups with gp120 Env protein co-administration during priming (groups T2 and T4), IgG response trajectories were similar between NYVAC vector and gp120 Env protein co-administration (group T2) and DNA vector and gp120 Env protein co-administration (group T4) after the month 3 and month 6 vaccinations (figure 3B).

For all groups, responses and magnitudes of response dropped between 2 weeks and 12 months after the month 6 vaccination, and no differences were noted in aggregate vaccine-matched response magnitudes between groups at 12 months after the month 6 vaccination (data not shown). No differences in vaccine-matched or V1/V2 IgG responses and magnitudes of response were seen between females and males (appendix pp 11, 12). The kinetics of IgG3 anti-Env responses to aggregate vaccine-matched and V1/V2 proteins were very similar to the kinetics of total IgG responses (appendix p 13).

The area under the IgG binding antibody response curve from month 0 to 12 months after the month 6 vaccination was significantly higher when gp120 Env protein was co-administered with DNA vector (group T4) than with DNA vector alone (group T3; p=0·0005 for the aggregate vaccine-matched response for T4 *vs* T3; figure 3C). A higher area under the IgG binding antibody response curve was seen for eight of the ten antigens tested; the exception was 96ZM65.1gp140 and the antigens in the vector such as gp41 and p24. The effect of co-administration of gp120 Env protein with the NYVAC vector did not differ with respect to the aggregate vaccine-matched response (p=0·25 for T2 *vs* T1); however, an increased area under the IgG binding antibody response curve was apparent for three of the ten antigens tested, including gp70_B.Case A_V1_V2 and AE.A244 V1V2 Tags/293F (figure 3C). Co-administration of gp120 Env protein with DNA vector priming (group T4) versus NYVAC vector priming (group T2) yielded a significantly higher area under the IgG binding antibody response curve for the aggregate vaccine-matched response and for three of the ten antigens tested (p=0·032 for T4 *vs* T2; figure 3C). Thus, priming with a DNA vector co-administered with gp120 Env protein (group T4) generated the most rapid, potent, and durable binding antibody responses among the four vaccine regimens tested.

For most antigens, IgA responses and magnitudes of response peaked 2 weeks after the month 6 vaccination for groups T1 and T3, and were generally low and constant over time for groups T2 and T4 (data not shown). In general, IgA responses were low, with the exception of responses to Con6 gp120/B, ConS gp140 CFI, MN gp120 gDneg/293F/mon, and Gag p24 at 2 weeks after the month 6 vaccination (data not shown). Aggregate vaccine-matched responses 2 weeks after the month 6 vaccination ranged from 26% (group T4) to 82% (group T3). Responses and magnitudes of response 2 weeks after the month 6 vaccination were generally lower in groups with gp120 Env protein co-administration during the priming phase (groups T2 and T4). Very few positive responses were seen 6 months after the month 6 vaccination.

**Figure 4:**
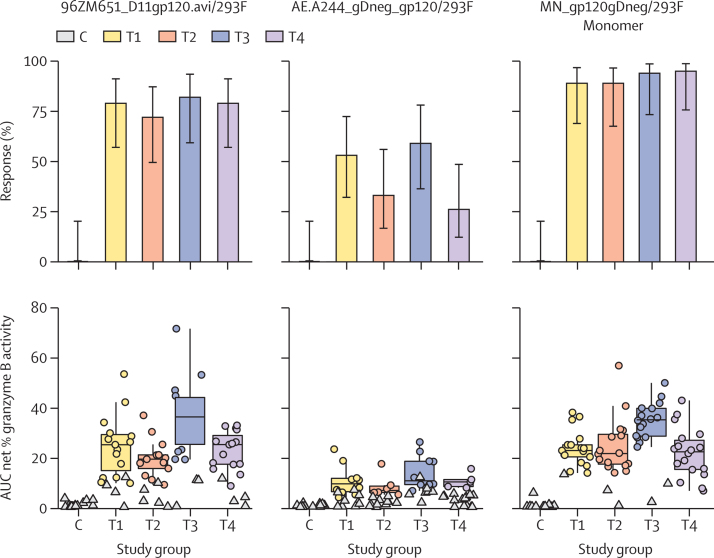
Antibody-dependent cell-mediated cytotoxicity Responses (upper panels) and AUC net percentage of granzyme B activity (lower panels) are shown for three different antigens in the study groups. The AUC was the non-parametric area under the net percentage granzyme B activity versus log_10_ (dilution) curve and was calculated using the trapezoidal rule. Circles show positive responses; negative responses are shown as triangles. Boxplots represent the distribution of positive responders only. Clade C gp120 was 96ZM651_D11gp120.avi/293F; clade AE gp120 was AE.A244_gDneg_gp120/293F; and clade B was MN_gp120gDneg/293F Monomer. As control, serum samples from the treated groups were incubated with target cells not coated with gp120. T1=two doses of NYVAC vector followed by two doses of NYVAC vector and gp120 Env protein. T2=four doses of NYVAC vector and gp120 Env protein. T3=two doses of DNA vector followed by two doses of NYVAC vector and gp120 Env protein. T4=two doses of DNA vector and gp120 Env protein followed by two doses of NYVAC vector and gp120 Env protein. C=placebo groups (C1–C4), comprising matched injections. AUC=area under the curve. Env=envelope.

Findings of the ADCC GTL assay showed that anti-Env responses to the antigens 96ZM65.1_Δ11gp120.avi/293F, AE.A244_gDneg_gp120/293F, and MN_gp120gDneg/293F Monomer at 2 weeks after the month 6 vaccination did not differ between groups (appendix p 14). However, the magnitudes of response to 96ZM65.1_Δ11gp120.avi/293F and MN_gp120gDneg/293F Monomer among positive responders were higher with DNA vector priming alone (group T3) compared with co-administration of DNA vector and gp120 Env protein (group T4; p=0·0023 for T3 *vs* T4 response to 96ZM65.1_Δ11gp120.avi/293F and p=0·0002 for T3 *vs* T4 response to MN_gp120gDneg/293F Monomer). DNA vector priming versus NYVAC vector priming (with or without gp120 Env protein co-administration) yielded similar responses (figure 4). For the 96ZM65.1_Δ11gp120.avi/293F and MN_gp120gDneg/293F Monomer antigens, magnitudes of response among positive responders were significantly higher for the DNA vector (group T3) versus NYVAC vector (group T1) primed groups (p=0·026 and p<0·0001, respectively). The peak net percentage granzyme B activity was ascertained as secondary readout for ADCC. The results obtained were consistent with the profiles of the AUC net percentage granzyme B activity (appendix p 14).

A global representation of the kinetics of the T-cell and antibody responses is shown in the appendix (p 15). The analysis clearly indicates that early antibody responses (including binding IgG Env, IgG3 V1/V2 aggregate vaccine-matched, and MN.3 neutralising antibody) are affected positively by co-administration during priming of gp120 Env protein with NYVAC vector (group T2) or DNA vector (group T4), with peak response and high magnitude present as early as 2 weeks after the month 1 vaccination. Antibody responses peaked at 2 weeks after the month 6 vaccination in the groups that did not receive gp120 Env protein during priming (groups T1 and T3). Antibody responses waned over time in all study groups, and no differences between groups were seen in this decline.

Comparative analyses of IgG responses 2 weeks after the month 6 vaccination were done using data from this study (HVTN 096, combining data across vaccine groups) and the studies RV144 and HVTN 100 (NCT02404311). HVTN 100 is a phase 1/2, randomised controlled double-blind trial in healthy volunteers at low risk of HIV infection. The vaccine regimen under assessment in HVTN 100 was composed of an ALVAC-HIV (vCP2438) vector expressing Env gp120 (subtype C 96ZM651), the transmembrane region of Env gp41, Gag, and protease (all subtype B HIV-1 LAI) plus bivalent subtype C gp120 and MF59 adjuvant.^33^ This vaccine regimen is under further assessment in a phase 2b efficacy trial (HVTN 702; NCT02968849).

**Figure 5:**
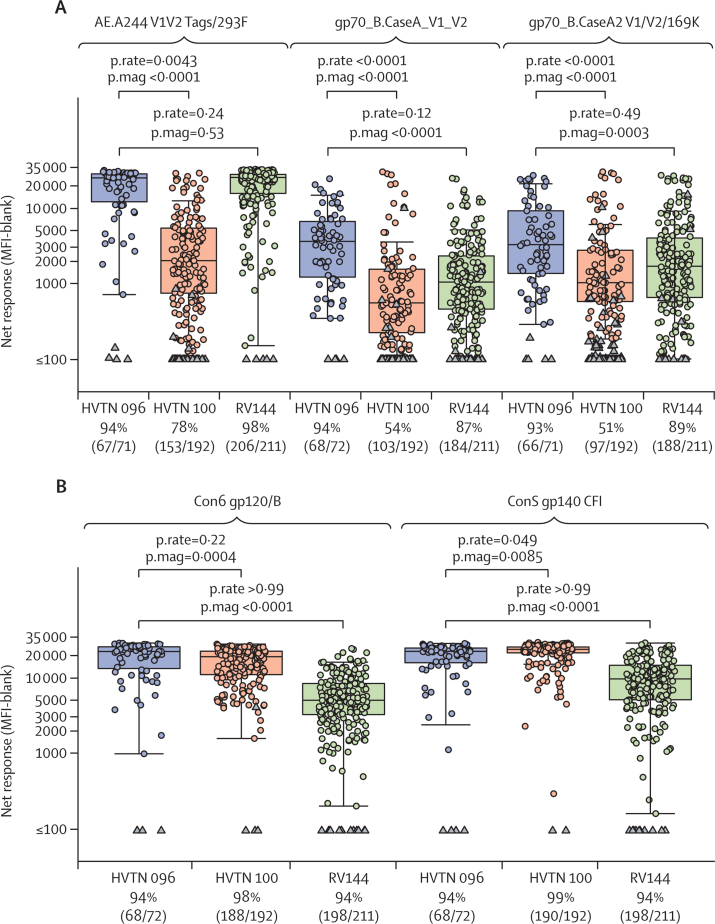
Cross-protocol comparisons of HVTN 096, RV144, and HVTN 100 studies (A) Comparison of V1/V2 antigens common across the three protocols at peak timepoints (with study groups of HVTN 096 pooled): AE.A244 V1V2 Tags 293F (matched), gp70_B CaseA_V1_V2 (not matched), and gp70_B CaseA2 V1/V2/169K (not matched). (B) Comparison of IgG responses to gp120 antigens across the three protocols at peak timepoints (with study groups of HVTN 096 pooled): Con 6 gp12/B and Con S gp140 CFI. Boxplots show the distribution of positive responses. Circles show positive responses; negative responses are shown as triangles. The proportion of positive responders at peak timepoints (2 weeks after the month 6 vaccination) are shown at the bottom of each panel. p.rate=p value for difference in response. p.mag=p value for difference in magnitude of response. HVTN=HIV Vaccine Trials Network. MFI=mean fluorescence intensity.

IgG binding antibody responses and magnitudes of responses to AE.A244 V1/V2 Tags293F, gp_70B.CaseA V1/V2, and gp_70B.CaseA2 V1/V2/169K were superior in HVTN 096 compared with HVTN 100 (figure 5). With respect to HVTN 096 versus RV144, no differences in response were recorded for the three V1/V2 proteins, but the magnitudes of responses to gp70_B.CaseA V1/V2 and gp70_B.CaseA2 V1/V2/169K were higher in HVTN 096 compared with RV144 (figure 5). With respect to binding IgG responses to Con6 gp120/B and ConS gp140 CFI, differences in response did not differ between vaccine regimens, but response magnitudes did. Comparing HVTN 096 and HVTN 100, higher response magnitudes to Con6 gp120/B were noted in HVTN 096 and higher ConS gp140 CFI responses were seen in HVTN 100. Comparing HVTN 096 and RV144, the magnitudes of the gp120 and gp140 responses were significantly higher in HVTN 096 (figure 5).

## Discussion

Our data indicate that early administration of gp120 Env protein leads to early elicitation of binding and neutralising immune responses, with no effect on T-cell responses. The overall area under the IgG binding antibody response curve with early administration of gp120 Env protein is higher and hence might offer enhanced efficacy as a vaccine regimen. The higher area under the IgG binding antibody response curve is more a result of earlier induction of antibodies and less an outcome of greater durability or overall magnitude of response.

Previous phase 1/2 clinical studies (eg, the EV01 trial^34^ and the EV02 trial^35^) have investigated the immunogenicity of NYVAC vector-based vaccine regimens in homologous or heterologous DNA vector-based priming and NYVAC vector-based boost combinations. In these studies, responses and magnitudes of responses for both CD4 and CD8 T cells were greatly increased in the regimens with a DNA vector-based prime and a NYVAC vector-based boost. It is important to underscore that both the NYVAC and DNA vector-based vaccines used in the EV02 trial^35^ and another trial (EV03) were different from those used in HVTN 096 with respect to the clade C subtype, the number of NYVAC vectors and DNA vectors used, and the optimisation of HIV immunogen expression. The immunogenicity results obtained in HVTN 096 are highly consistent with those obtained in the EV01 trial^34^ and EV02 trial^35^ and indicate that, in study groups not containing proteins in the prime (T1 and T3), peak responses and magnitudes of response occurred 2 weeks after the month 3 vaccination for CD4 T cells and 2 weeks after the month 6 vaccination for CD8 T cells. CD4 T-cell responses were durable and remained unchanged in both responses and magnitudes of response by 6 months after the month 6 vaccination. These results, therefore, further confirm the superiority of heterologous prime and boost DNA and NYVAC vector-based regimens compared with homologous prime and boost NYVAC vector-based regimens.

The RV144 study identified binding IgG antibodies directed at conserved regions of the V1/V2 loop, and antibodies mediating ADCC in the presence of low plasma IgA, as immune correlates of reduced risk of HIV infection.^11^ Of note, the highest protection from infection was seen during the first year of follow-up, then protection waned over time, which was associated with a substantial drop in antibody responses correlating with protection.^12^ Based on the results from RV144, it is important to increase the levels of protection seen during the first year and to maintain a durable antibody response over time so we can substantially improve the modest protection recorded at 42 months after the first vaccination.

HVTN 096 was designed to ascertain whether gp120 Env protein co-administration at priming could induce rapid and durable generation of the antibody responses that were shown to correlate with protection against infection in RV144. In HVTN 096, we used the same bivalent gp120 Env protein vaccine as was used in RV144. Use of a mismatched clade Env protein vaccine compared with clade C immunogens expressed in the NYVAC and DNA vector-based vaccines offered the opportunity to assess the induction of cross-clade immune responses. The vaccine regimens tested in HVTN 096 induced broad cross-clade antibody responses.

Co-administration of gp120 Env protein at priming resulted in rapid and potent generation of the antibody responses recorded in RV144. Binding IgG Env antibody responses, including anti-V1/V2, were detected 2 weeks after the second dose of gp120 Env protein co-administration (with either the NYVAC or DNA vector) in almost 100% of participants, and levels of antibodies were close to peak amounts that were generally seen 2 weeks after the month 3 vaccination. Antibody responses and magnitudes of responses peaked 2 weeks after the month 6 vaccination in study groups without gp120 Env protein co-administration at priming. Rapid and potent generation of antibody responses occurs also when gp120 Env proteins are administered as monovalent vaccine.^1^, ^2^ Therefore, the rapid induction of antibody responses seen in HVTN 096 most likely results from early co-administration of gp120 Env protein, with no evidence seen of an interaction between gp120 Env protein and DNA or NYVAC vectors.

Responses for binding IgG antibodies directed to V1/V2 were similar between HVTN 096 and RV144 at 2 weeks after the month 6 vaccination, whereas the magnitudes of responses were superior in HVTN 096 for two of three V1/V2 antigens. However, both responses and magnitudes of responses of binding IgG antibodies directed to V1/V2 were superior in HVTN 096 versus HVTN 100. These findings confirm previous observations of the immune response advantage of NYVAC vector-based vaccines versus ALVAC vector-based vaccines administered with Env protein in non-human primates.^36^ However, the different gp120 proteins and formulations used in HVTN 096 and HVTN 100 might have also had an effect on the differences seen in antibody responses. Binding IgG antibody responses to gp120 or gp140 proteins were similar between HVTN 096, HVTN 100, and RV144, whereas the magnitude of these responses was generally superior in HVTN 096.

Binding IgG Env antibody responses to aggregate vaccine-matched Env proteins and to V1/V2 dropped over time in all study groups in HVTN 096. However, binding antibody responses were detected 6 months after the month 6 vaccination in many individuals in all study groups: responses ranged from 58% to 81% for aggregate vaccine-matched Env proteins and from 47% to 58% for AE.A244 V1V2 Tags/293F. Vaccine-induced Env V1/V2 IgG3 responses were associated with lower risk of infection in RV144.^13^ IgG3 responses to aggregate vaccine-matched and V1/V2 Env proteins were also present in all vaccine study groups in HVTN 096 and had kinetics similar to total IgG responses. Consistent with the findings of RV144, IgG3 responses in HVTN 096 waned over time. ADCC activity against two of three vaccine-matched Env proteins was detected in many (70–95%) individuals 2 weeks after the month 6 vaccination.

Overall, the four vaccine regimens assessed in HVTN 096 were all immunogenic, with T-cell responses and a subset of antibody responses that were higher, on average, in the regimens containing the DNA vector. Co-administration of Env protein at priming is not only associated with rapid generation of protective antibody responses but also has better antibody response coverage over a period of 18 months compared with regimens without protein co-administration at priming. Although early Env protein co-administration could be associated with improved protection from infection during the first year of vaccination, the decline in responses and magnitudes of responses over time is not prevented satisfactorily by co-administration of Env protein at priming. Therefore, additional vaccinations, improved formulation of Env proteins with more potent adjuvants, or both need to be tested to induce durable antibody responses.

The generalisability of the immune correlates identified in the RV144 study has not yet been confirmed in other efficacy trials. Furthermore, since the vaccines analysed in the HVTN 096 study are different to those assessed in RV144, the protective capacity of the vaccine-induced immune responses in HVTN 096 remains unknown. The results from HVTN 096 provide the basis for designing new studies assessing the benefit of early Env protein co-administration with other candidate vaccines or improved formulations and underscore the importance of further developing DNA and poxvirus vector-based vaccine regimens.

**This online publication has been corrected. The corrected version first appeared at thelancet.com/hiv on January 9, 2020**
