# Correction to *Lancet HIV* 2019; 6: e737–49

**DOI:** 10.1016/S2352-3018(20)30002-3

**Published:** 2020-01-09

*Pantaleo G, Janes H, Karuna S, et al. Safety and immunogenicity of a multivalent HIV vaccine comprising envelope protein with either DNA or NYVAC vectors (HVTN 096): a phase 1b, double-blind, placebo-controlled trial.* Lancet HIV *2019;* 6: *e737–49*—In this Article, the label and data for the study group T4 in the right hand panel of figure 3C were presented incorrectly. This correction was made on Jan 9, 2020.

