# Supplementary material for: Safety and immunogenicity of a multivalent HIV vaccine comprising envelope protein with either DNA or NYVAC vectors (HVTN 096): a phase 1b, double-blind, placebo-controlled trial
Source: Lancet HIV. 2019 Oct 7;6(11):e737–49. doi: 10.1016/S2352-3018(19)30262-0 (PMC7156919; doi:10.1016/S2352-3018(19)30262-0)
Supplement: Supplementary appendix [file mmc1.pdf]

THE LANCET
HIV

Supplementary appendix

This appendix formed part of the original submission and has been peer reviewed. We post it as supplied by the authors.

Supplement to: Pantaleo G, Janes H, Karuna S, et al. Safety and immunogenicity of a multivalent HIV vaccine comprising envelope protein with either DNA or NYVAC vectors (HVTN 096): a phase 1b, double-blind, placebo-controlled trial. *Lancet HIV* 2019; published online Oct 7. https://doi.org/10.1016/S2352-3018(19)30262-0.

Supplementary Material for

Safety and immunogenicity of a multivalent HIV vaccine comprising Env protein with either DNA or NYVAC vectors (HVTN 096): a phase 1b, double-blind, placebo-controlled trial

Pantaleo et al.

Supplementary Table 1. Baseline demographics and vaccinations received by treatment group in HVTN 096.

	Control (C1-4) (n = 16)	T1: N/N/NA/NA (n = 20)	T2: NA/NA/NA/NA (n = 20)	T3: D/D/NA/NA (n = 20)	T4: DA/DA/NA/NA (n = 20)	Overall (n = 96)
Sex						
Male	8 (50%)	10 (50%)	10 (50%)	9 (45%)	10 (50%)	47 (49%)
Female	8 (50%)	10 (50%)	10 (50%)	11 (55%)	10 (50%)	49 (51%)
Race/ethnicity*						
White – NH	13 (81%)	15 (75%)	14 (70%)	15 (75%)	15 (75%)	72 (75%)
Black/AA - NH	0 (0%)	1 (5%)	1 (5%)	0 (0%)	0 (0%)	2 (2%)
Hispanic	3 (19%)	4 (20%)	5 (25%)	4 (20%)	4 (20%)	20 (21%)
Asian	0 (0%)	0 (0%)	0 (0%)	1 (5%)	0 (0%)	1 (1%)
Other	0 (0%)	0 (0%)	0 (0%)	0 (0%)	1 (5%)	1 (1%)
Age (Years)						
18-20	2 (13%)	0 (0%)	1 (5%)	2 (10%)	4 (20%)	9 (9%)
21-30	11 (69%)	11 (55%)	14 (70%)	13 (65%)	11 (55%)	60 (63%)
31-40	1 (6%)	7 (35%)	4 (20%)	4 (20%)	3 (15%)	19 (20%)
41-50	2 (13%)	2 (10%)	1 (5%)	1 (5%)	2 (10%)	8 (8%)
Vaccination Frequencies						
Week 0	16 (100%)	20 (100%)	20 (100%)	20 (100%)	20 (100%)	96 (100%)
Week 4	16 (100%)	19 (95%)	20 (100%)	20 (100%)	20 (100%)	95 (99%)
Week 12	16 (100%)	18 (90%)	18 (90%)	20 (100%)	19 (95%)	91 (95%)
Week 24	15 (94%)	18 (90%)	16 (80%)	16 (80%)	18 (90%)	83 (86%)

*AA, African American. NH, non-Hispanic

Supplemental Table 2. Summary of cumulative adverse events by treatment. Adverse events are listed by MedDRA body system and severity grade, and by preferred term and relationship to study product, by decreasing frequency.

		Control (C1-4)		T1: N/N/NA/NA		T2: NA/NA/NA/NA		T3: D/D/NA/NA		T4: DA/DA/NA/NA		Total	
		(n = 16)		(n = 20)		(n = 20)		(n = 20)		(n = 20)		(N = 96)	
		n	(%)	n	(%)	n	(%)	n	(%)	n	(%)	n	(%)
Body System / Severity*	**Participants with one or more AEs**												
	Mild	4	(25.0%)	5	(25.0%)	8	(40.0%)	8	(40.0%)	7	(35.0%)	32	(33.3%)
	Moderate	9	(56.3%)	14	(70.0%)	9	(45.0%)	7	(35.0%)	12	(60.0%)	51	(53.1%)
	Severe	2	(12.5%)	1	(5.0%)	3	(15.0%)	1	(5.0%)	0	(0.0%)	7	(7.3%)
	Potentially Life-Threatening	0	(0.0%)	0	(0.0%)	0	(0.0%)	0	(0.0%)	0	(0.0%)	0	(0.0%)
	Death	0	(0.0%)	0	(0.0%)	0	(0.0%)	1	(5.0%)	0	(0.0%)	1	(1.0%)
	Total	15	(93.8%)	20	(100.0%)	20	(100.0%)	17	(85.0%)	19	(95.0%)	91	(94.8%)
	**Infections and infestations**												
	Mild	12	(75.0%)	10	(50.0%)	12	(60.0%)	7	(35.0%)	10	(50.0%)	51	(53.1%)
	Moderate	2	(12.5%)	7	(35.0%)	5	(25.0%)	6	(30.0%)	5	(25.0%)	25	(26.0%)
	Severe	1	(6.3%)	0	(0.0%)	0	(0.0%)	0	(0.0%)	0	(0.0%)	1	(1.0%)
	Potentially Life-Threatening	0	(0.0%)	0	(0.0%)	0	(0.0%)	0	(0.0%)	0	(0.0%)	0	(0.0%)
	Death	0	(0.0%)	0	(0.0%)	0	(0.0%)	0	(0.0%)	0	(0.0%)	0	(0.0%)
	Total	15	(93.8%)	17	(85.0%)	17	(85.0%)	13	(65.0%)	15	(75.0%)	77	(80.2%)
	**General disorders and administration site conditions**												
	Mild	6	(37.5%)	3	(15.0%)	9	(45.0%)	8	(40.0%)	9	(45.0%)	35	(36.5%)
	Moderate	0	(0.0%)	0	(0.0%)	1	(5.0%)	1	(5.0%)	2	(10.0%)	4	(4.2%)
	Severe	0	(0.0%)	0	(0.0%)	0	(0.0%)	0	(0.0%)	0	(0.0%)	0	(0.0%)
	Potentially Life-Threatening	0	(0.0%)	0	(0.0%)	0	(0.0%)	0	(0.0%)	0	(0.0%)	0	(0.0%)
	Death	0	(0.0%)	0	(0.0%)	0	(0.0%)	0	(0.0%)	0	(0.0%)	0	(0.0%)
	Total	6	(37.5%)	3	(15.0%)	10	(50.0%)	9	(45.0%)	11	(55.0%)	39	(40.6%)
	**Gastrointestinal disorders**												
	Mild	2	(12.5%)	3	(15.0%)	3	(15.0%)	2	(10.0%)	2	(10.0%)	12	(12.5%)
	Moderate	2	(12.5%)	3	(15.0%)	1	(5.0%)	2	(10.0%)	2	(10.0%)	10	(10.4%)
	Severe	0	(0.0%)	0	(0.0%)	1	(5.0%)	0	(0.0%)	0	(0.0%)	1	(1.0%)
	Potentially Life-Threatening	0	(0.0%)	0	(0.0%)	0	(0.0%)	0	(0.0%)	0	(0.0%)	0	(0.0%)
	Death	0	(0.0%)	0	(0.0%)	0	(0.0%)	0	(0.0%)	0	(0.0%)	0	(0.0%)
	Total	4	(25.0%)	6	(30.0%)	5	(25.0%)	4	(20.0%)	4	(20.0%)	23	(24.0%)
	**Injury, poisoning and procedural complications**												
	Mild	1	(6.3%)	2	(10.0%)	4	(20.0%)	0	(0.0%)	1	(5.0%)	8	(8.3%)
	Moderate	2	(12.5%)	3	(15.0%)	2	(10.0%)	2	(10.0%)	2	(10.0%)	11	(11.5%)
	Severe	0	(0.0%)	0	(0.0%)	1	(5.0%)	0	(0.0%)	0	(0.0%)	1	(1.0%)
	Potentially Life-Threatening	0	(0.0%)	0	(0.0%)	0	(0.0%)	0	(0.0%)	0	(0.0%)	0	(0.0%)
	Death	0	(0.0%)	0	(0.0%)	0	(0.0%)	1	(5.0%)	0	(0.0%)	1	(1.0%)
	Total	3	(18.8%)	5	(25.0%)	7	(35.0%)	3	(15.0%)	3	(15.0%)	21	(21.9%)
	**Nervous system disorders**												
	Mild	2	(12.5%)	0	(0.0%)	6	(30.0%)	3	(15.0%)	3	(15.0%)	14	(14.6%)
	Moderate	1	(6.3%)	2	(10.0%)	0	(0.0%)	0	(0.0%)	2	(10.0%)	5	(5.2%)

Severe	0	(0.0%)	0	(0.0%)	0	(0.0%)	0	(0.0%)	0	(0.0%)	0	(0.0%)
Potentially Life-Threatening	0	(0.0%)	0	(0.0%)	0	(0.0%)	0	(0.0%)	0	(0.0%)	0	(0.0%)
Death	0	(0.0%)	0	(0.0%)	0	(0.0%)	0	(0.0%)	0	(0.0%)	0	(0.0%)
Total	3	(18.8%)	2	(10.0%)	6	(30.0%)	3	(15.0%)	5	(25.0%)	19	(19.8%)
**Investigations**												
Mild	2	(12.5%)	2	(10.0%)	2	(10.0%)	0	(0.0%)	6	(30.0%)	12	(12.5%)
Moderate	0	(0.0%)	1	(5.0%)	1	(5.0%)	0	(0.0%)	0	(0.0%)	2	(2.1%)
Severe	0	(0.0%)	0	(0.0%)	0	(0.0%)	0	(0.0%)	0	(0.0%)	0	(0.0%)
Potentially Life-Threatening	0	(0.0%)	0	(0.0%)	0	(0.0%)	0	(0.0%)	0	(0.0%)	0	(0.0%)
Death	0	(0.0%)	0	(0.0%)	0	(0.0%)	0	(0.0%)	0	(0.0%)	0	(0.0%)
Total	2	(12.5%)	3	(15.0%)	3	(15.0%)	0	(0.0%)	6	(30.0%)	14	(14.6%)
**Musculoskeletal and connective tissue disorders**												
Mild	1	(6.3%)	0	(0.0%)	4	(20.0%)	2	(10.0%)	1	(5.0%)	8	(8.3%)
Moderate	0	(0.0%)	2	(10.0%)	2	(10.0%)	0	(0.0%)	2	(10.0%)	6	(6.3%)
Severe	0	(0.0%)	0	(0.0%)	0	(0.0%)	0	(0.0%)	0	(0.0%)	0	(0.0%)
Potentially Life-Threatening	0	(0.0%)	0	(0.0%)	0	(0.0%)	0	(0.0%)	0	(0.0%)	0	(0.0%)
Death	0	(0.0%)	0	(0.0%)	0	(0.0%)	0	(0.0%)	0	(0.0%)	0	(0.0%)
Total	1	(6.3%)	2	(10.0%)	6	(30.0%)	2	(10.0%)	3	(15.0%)	14	(14.6%)
**Blood and lymphatic system disorders**												
Mild	0	(0.0%)	6	(30.0%)	3	(15.0%)	0	(0.0%)	4	(20.0%)	13	(13.5%)
Moderate	0	(0.0%)	0	(0.0%)	0	(0.0%)	0	(0.0%)	0	(0.0%)	0	(0.0%)
Severe	0	(0.0%)	0	(0.0%)	0	(0.0%)	0	(0.0%)	0	(0.0%)	0	(0.0%)
Potentially Life-Threatening	0	(0.0%)	0	(0.0%)	0	(0.0%)	0	(0.0%)	0	(0.0%)	0	(0.0%)
Death	0	(0.0%)	0	(0.0%)	0	(0.0%)	0	(0.0%)	0	(0.0%)	0	(0.0%)
Total	0	(0.0%)	6	(30.0%)	3	(15.0%)	0	(0.0%)	4	(20.0%)	13	(13.5%)
**Psychiatric disorders**												
Mild	0	(0.0%)	1	(5.0%)	4	(20.0%)	2	(10.0%)	0	(0.0%)	7	(7.3%)
Moderate	2	(12.5%)	0	(0.0%)	1	(5.0%)	0	(0.0%)	1	(5.0%)	4	(4.2%)
Severe	0	(0.0%)	1	(5.0%)	0	(0.0%)	1	(5.0%)	0	(0.0%)	2	(2.1%)
Potentially Life-Threatening	0	(0.0%)	0	(0.0%)	0	(0.0%)	0	(0.0%)	0	(0.0%)	0	(0.0%)
Death	0	(0.0%)	0	(0.0%)	0	(0.0%)	0	(0.0%)	0	(0.0%)	0	(0.0%)
Total	2	(12.5%)	2	(10.0%)	5	(25.0%)	3	(15.0%)	1	(5.0%)	13	(13.5%)
**Skin and subcutaneous tissue disorders**												
Mild	1	(6.3%)	1	(5.0%)	3	(15.0%)	1	(5.0%)	2	(10.0%)	8	(8.3%)
Moderate	2	(12.5%)	0	(0.0%)	1	(5.0%)	0	(0.0%)	0	(0.0%)	3	(3.1%)
Severe	0	(0.0%)	0	(0.0%)	0	(0.0%)	0	(0.0%)	0	(0.0%)	0	(0.0%)
Potentially Life-Threatening	0	(0.0%)	0	(0.0%)	0	(0.0%)	0	(0.0%)	0	(0.0%)	0	(0.0%)
Death	0	(0.0%)	0	(0.0%)	0	(0.0%)	0	(0.0%)	0	(0.0%)	0	(0.0%)
Total	3	(18.8%)	1	(5.0%)	4	(20.0%)	1	(5.0%)	2	(10.0%)	11	(11.5%)
**Respiratory, thoracic and mediastinal disorders**												
Mild	1	(6.3%)	1	(5.0%)	5	(25.0%)	1	(5.0%)	0	(0.0%)	8	(8.3%)
Moderate	0	(0.0%)	0	(0.0%)	0	(0.0%)	1	(5.0%)	1	(5.0%)	2	(2.1%)
Severe	0	(0.0%)	0	(0.0%)	0	(0.0%)	0	(0.0%)	0	(0.0%)	0	(0.0%)
Potentially Life-Threatening	0	(0.0%)	0	(0.0%)	0	(0.0%)	0	(0.0%)	0	(0.0%)	0	(0.0%)
Death	0	(0.0%)	0	(0.0%)	0	(0.0%)	0	(0.0%)	0	(0.0%)	0	(0.0%)
Total	1	(6.3%)	1	(5.0%)	5	(25.0%)	2	(10.0%)	1	(5.0%)	10	(10.4%)
**Metabolism and nutrition disorders**												
Mild	1	(6.3%)	1	(5.0%)	0	(0.0%)	1	(5.0%)	1	(5.0%)	4	(4.2%)
Moderate	0	(0.0%)	1	(5.0%)	1	(5.0%)	0	(0.0%)	1	(5.0%)	3	(3.1%)
Severe	0	(0.0%)	0	(0.0%)	1	(5.0%)	0	(0.0%)	0	(0.0%)	1	(1.0%)

Potentially Life-Threatening	0	(0.0%)	0	(0.0%)	0	(0.0%)	0	(0.0%)	0	(0.0%)	0	(0.0%)
Death	0	(0.0%)	0	(0.0%)	0	(0.0%)	0	(0.0%)	0	(0.0%)	0	(0.0%)
Total	1	(6.3%)	2	(10.0%)	2	(10.0%)	1	(5.0%)	2	(10.0%)	8	(8.3%)
**Eye disorders**												
Mild	0	(0.0%)	2	(10.0%)	1	(5.0%)	0	(0.0%)	1	(5.0%)	4	(4.2%)
Moderate	0	(0.0%)	1	(5.0%)	0	(0.0%)	0	(0.0%)	0	(0.0%)	1	(1.0%)
Severe	0	(0.0%)	0	(0.0%)	0	(0.0%)	0	(0.0%)	0	(0.0%)	0	(0.0%)
Potentially Life-Threatening	0	(0.0%)	0	(0.0%)	0	(0.0%)	0	(0.0%)	0	(0.0%)	0	(0.0%)
Death	0	(0.0%)	0	(0.0%)	0	(0.0%)	0	(0.0%)	0	(0.0%)	0	(0.0%)
Total	0	(0.0%)	3	(15.0%)	1	(5.0%)	0	(0.0%)	1	(5.0%)	5	(5.2%)
**Cardiac disorders**												
Mild	0	(0.0%)	0	(0.0%)	1	(5.0%)	0	(0.0%)	1	(5.0%)	2	(2.1%)
Moderate	0	(0.0%)	1	(5.0%)	1	(5.0%)	0	(0.0%)	0	(0.0%)	2	(2.1%)
Severe	0	(0.0%)	0	(0.0%)	0	(0.0%)	0	(0.0%)	0	(0.0%)	0	(0.0%)
Potentially Life-Threatening	0	(0.0%)	0	(0.0%)	0	(0.0%)	0	(0.0%)	0	(0.0%)	0	(0.0%)
Death	0	(0.0%)	0	(0.0%)	0	(0.0%)	0	(0.0%)	0	(0.0%)	0	(0.0%)
Total	0	(0.0%)	1	(5.0%)	2	(10.0%)	0	(0.0%)	1	(5.0%)	4	(4.2%)
**Ear and labyrinth disorders**												
Mild	0	(0.0%)	0	(0.0%)	0	(0.0%)	1	(5.0%)	1	(5.0%)	2	(2.1%)
Moderate	1	(6.3%)	0	(0.0%)	1	(5.0%)	0	(0.0%)	0	(0.0%)	2	(2.1%)
Severe	0	(0.0%)	0	(0.0%)	0	(0.0%)	0	(0.0%)	0	(0.0%)	0	(0.0%)
Potentially Life-Threatening	0	(0.0%)	0	(0.0%)	0	(0.0%)	0	(0.0%)	0	(0.0%)	0	(0.0%)
Death	0	(0.0%)	0	(0.0%)	0	(0.0%)	0	(0.0%)	0	(0.0%)	0	(0.0%)
Total	1	(6.3%)	0	(0.0%)	1	(5.0%)	1	(5.0%)	1	(5.0%)	4	(4.2%)
**Hepatobiliary disorders**												
Mild	0	(0.0%)	0	(0.0%)	1	(5.0%)	0	(0.0%)	1	(5.0%)	2	(2.1%)
Moderate	0	(0.0%)	0	(0.0%)	0	(0.0%)	0	(0.0%)	0	(0.0%)	0	(0.0%)
Severe	0	(0.0%)	0	(0.0%)	0	(0.0%)	0	(0.0%)	0	(0.0%)	0	(0.0%)
Potentially Life-Threatening	0	(0.0%)	0	(0.0%)	0	(0.0%)	0	(0.0%)	0	(0.0%)	0	(0.0%)
Death	0	(0.0%)	0	(0.0%)	0	(0.0%)	0	(0.0%)	0	(0.0%)	0	(0.0%)
Total	0	(0.0%)	0	(0.0%)	1	(5.0%)	0	(0.0%)	1	(5.0%)	2	(2.1%)
**Neoplasms benign, malignant and unspecified (incl cysts and polyps)**												
Mild	0	(0.0%)	0	(0.0%)	0	(0.0%)	1	(5.0%)	0	(0.0%)	1	(1.0%)
Moderate	0	(0.0%)	1	(5.0%)	0	(0.0%)	0	(0.0%)	0	(0.0%)	1	(1.0%)
Severe	0	(0.0%)	0	(0.0%)	0	(0.0%)	0	(0.0%)	0	(0.0%)	0	(0.0%)
Potentially Life-Threatening	0	(0.0%)	0	(0.0%)	0	(0.0%)	0	(0.0%)	0	(0.0%)	0	(0.0%)
Death	0	(0.0%)	0	(0.0%)	0	(0.0%)	0	(0.0%)	0	(0.0%)	0	(0.0%)
Total	0	(0.0%)	1	(5.0%)	0	(0.0%)	1	(5.0%)	0	(0.0%)	2	(2.1%)
**Renal and urinary disorders**												
Mild	0	(0.0%)	0	(0.0%)	0	(0.0%)	1	(5.0%)	0	(0.0%)	1	(1.0%)
Moderate	0	(0.0%)	1	(5.0%)	0	(0.0%)	0	(0.0%)	0	(0.0%)	1	(1.0%)
Severe	0	(0.0%)	0	(0.0%)	0	(0.0%)	0	(0.0%)	0	(0.0%)	0	(0.0%)
Potentially Life-Threatening	0	(0.0%)	0	(0.0%)	0	(0.0%)	0	(0.0%)	0	(0.0%)	0	(0.0%)
Death	0	(0.0%)	0	(0.0%)	0	(0.0%)	0	(0.0%)	0	(0.0%)	0	(0.0%)
Total	0	(0.0%)	1	(5.0%)	0	(0.0%)	1	(5.0%)	0	(0.0%)	2	(2.1%)
**Reproductive system and breast disorders**												
Mild	0	(0.0%)	1	(5.0%)	0	(0.0%)	0	(0.0%)	1	(5.0%)	2	(2.1%)
Moderate	0	(0.0%)	0	(0.0%)	0	(0.0%)	0	(0.0%)	0	(0.0%)	0	(0.0%)
Severe	0	(0.0%)	0	(0.0%)	0	(0.0%)	0	(0.0%)	0	(0.0%)	0	(0.0%)
Potentially Life-Threatening	0	(0.0%)	0	(0.0%)	0	(0.0%)	0	(0.0%)	0	(0.0%)	0	(0.0%)

	Death	0	(0.0%)	0	(0.0%)	0	(0.0%)	0	(0.0%)	0	(0.0%)	0	(0.0%)
	Total	0	(0.0%)	1	(5.0%)	0	(0.0%)	0	(0.0%)	1	(5.0%)	2	(2.1%)
	**Congenital, familial and genetic disorders**												
	Mild	0	(0.0%)	0	(0.0%)	0	(0.0%)	0	(0.0%)	0	(0.0%)	0	(0.0%)
	Moderate	0	(0.0%)	0	(0.0%)	0	(0.0%)	0	(0.0%)	0	(0.0%)	0	(0.0%)
	Severe	1	(6.3%)	0	(0.0%)	0	(0.0%)	0	(0.0%)	0	(0.0%)	1	(1.0%)
	Potentially Life-Threatening	0	(0.0%)	0	(0.0%)	0	(0.0%)	0	(0.0%)	0	(0.0%)	0	(0.0%)
	Death	0	(0.0%)	0	(0.0%)	0	(0.0%)	0	(0.0%)	0	(0.0%)	0	(0.0%)
	Total	1	(6.3%)	0	(0.0%)	0	(0.0%)	0	(0.0%)	0	(0.0%)	1	(1.0%)
	**Endocrine disorders**												
	Mild	0	(0.0%)	0	(0.0%)	0	(0.0%)	0	(0.0%)	1	(5.0%)	1	(1.0%)
	Moderate	0	(0.0%)	0	(0.0%)	0	(0.0%)	0	(0.0%)	0	(0.0%)	0	(0.0%)
	Severe	0	(0.0%)	0	(0.0%)	0	(0.0%)	0	(0.0%)	0	(0.0%)	0	(0.0%)
	Potentially Life-Threatening	0	(0.0%)	0	(0.0%)	0	(0.0%)	0	(0.0%)	0	(0.0%)	0	(0.0%)
	Death	0	(0.0%)	0	(0.0%)	0	(0.0%)	0	(0.0%)	0	(0.0%)	0	(0.0%)
	Total	0	(0.0%)	0	(0.0%)	0	(0.0%)	0	(0.0%)	1	(5.0%)	1	(1.0%)
	**Social circumstances**												
	Mild	1	(6.3%)	0	(0.0%)	0	(0.0%)	0	(0.0%)	0	(0.0%)	1	(1.0%)
	Moderate	0	(0.0%)	0	(0.0%)	0	(0.0%)	0	(0.0%)	0	(0.0%)	0	(0.0%)
	Severe	0	(0.0%)	0	(0.0%)	0	(0.0%)	0	(0.0%)	0	(0.0%)	0	(0.0%)
	Potentially Life-Threatening	0	(0.0%)	0	(0.0%)	0	(0.0%)	0	(0.0%)	0	(0.0%)	0	(0.0%)
	Death	0	(0.0%)	0	(0.0%)	0	(0.0%)	0	(0.0%)	0	(0.0%)	0	(0.0%)
	Total	1	(6.3%)	0	(0.0%)	0	(0.0%)	0	(0.0%)	0	(0.0%)	1	(1.0%)
Preferred Term / Relationship to Product	**Participants with one or more AEs**	1	(6.3%)	5	(25.0%)	6	(30.0%)	1	(5.0%)	3	(15.0%)	16	(16.7%)
	Lymphadenopathy	0	(0.0%)	5	(25.0%)	3	(15.0%)	0	(0.0%)	1	(5.0%)	9	(9.4%)
	Hypoaesthesia	0	(0.0%)	0	(0.0%)	1	(5.0%)	0	(0.0%)	1	(5.0%)	2	(2.1%)
	Dizziness	0	(0.0%)	0	(0.0%)	0	(0.0%)	1	(5.0%)	0	(0.0%)	1	(1.0%)
	Injection site pruritus	0	(0.0%)	0	(0.0%)	0	(0.0%)	0	(0.0%)	1	(5.0%)	1	(1.0%)
	Palpitations	0	(0.0%)	0	(0.0%)	1	(5.0%)	0	(0.0%)	0	(0.0%)	1	(1.0%)
	Photosensitivity reaction	1	(6.3%)	0	(0.0%)	0	(0.0%)	0	(0.0%)	0	(0.0%)	1	(1.0%)
	Rash maculo-papular	0	(0.0%)	0	(0.0%)	1	(5.0%)	0	(0.0%)	0	(0.0%)	1	(1.0%)
*Participants are counted at most once for each MEDdra body system, under the highest severity they experienced.													

Supplemental Table 3. Details of antigens used in Intracellular Cytokine Staining (ICS), Binding Antibody Multiplex Assays (BAMA), Neutralizing Antibody (nAb), and Antibody Dependent Cellular Cytotoxicity (ADCC).

Assay	Antigen Class	Antigen	Viral Strain Information: Subtype.Country.Year.Stage*	Justification for selection
ICS	-	PTE_{g} - global Potential T-cell Epitope peptide pools representing 15-mer peptides (Gag-1-PTEg, Gag-2-PTEg, Pol-1-PTEg, Pol-2-PTEg, Pol-3-PTEg, Env-1-PTEg, Env-2-PTEg, Env-3-PTEg, and Nef-PTEg)	[Global potential T cell epitopes were selected based on sequences of circulating Group M strains in the Los Alamos database and these were combined into several pools based on frequency of occurrence in the database with the more frequent epitopes in the first pools]	The PTE peptide pools serve as a common standardized panel of HIV-1 peptides for evaluating responses across diverse vaccine candidates
nAb	EPV**	BaL.26	B.US.85.6	Tier 1A phenotype
	EPV**	MN.3	B.US.84.6	Tier 1A phenotype
	EPV**	SF162.LS	B.US.89.6	Tier 1A phenotype
	EPV**	MW965.26	C.MW.93.6	Tier 1A phenotype
	EPV**	NP03.13	CRF01_AE.TH.95.xx	Tier 1A phenotype
	EPV**	TH023.6	CRF01_AE.TH.92.6	Tier 1A phenotype
	EPV**	96ZM651.2	C.ZM.96.6	Env strain in the priming vectors
BAMA	Vaccine-matched antigens	96ZM65.1 gp140	C.ZM.96.6	Env strain in the priming vectors
		A244 gp120 gDneg/293F/mon	CRF01_AE.TH.90.6	Env strain in the protein boost
		MN gp120 gDneg/293F/mon	B.US.84.6	Env strain in the protein boost
	V1/V2 antigens	AE.A244 V1V2 Tags/293F	CRF01_AE.TH.90.6	RV144 V2 correlate
		gp70_B.CaseA_V1_V2	B.US.88.6	RV144 V2 correlate
		gp70_B.CaseA2 V1/V2/169K	B.US.88.6	RV144 V2 correlate
	Additional antigens	ConS gp140 CFI	[Consensus]	Standard immunogenicity Env (sensitive for vaccine elicited antibodies and to compare across protocols)
		Con6 gp120/B	[Consensus]	Standard immunogenicity Env (sensitive for vaccine elicited antibodies and to compare across protocols)
		gp41	B.xx.xx.xx	Standard immunogenicity Env (sensitive for vaccine elicited antibodies and to compare across protocols)
		p24	B.xx.xx.xx [Gag]	Vector: Standard immunogenicity Gag (sensitive for vaccine elicited antibodies and to compare across protocols)
ADCC	Vaccine-matched antigens	96ZM65.1_Δ11gp120.avi/293F	C.ZM.96.6	Recombinant HIV-1 gp120 glycoproteins selected to match the sequences of the HIV-1 envelopes included in the vaccine regimen. The 96ZM65.1 recombinant gp120 was included in the recombinant DNA and MVA and produced in the 293F cell line without the first 11 amino acid at the N-terminus to improve its immunogenicity. The A244 and MN recombinant gp120 were included in the AIDSVAX B/E product, and were produced as monomeric proteins in the 293F cell line in absence of the HSV gD (gDneg) protein tag originally inserted to improve their purification.
		AE.A244_gDneg_gp120/293F	CRF01_AE.TH.90.6	
		MN_gp120gDneg/293F Monomer	B.US.84.6	

*Subtype is denoted by a capital letter; country of origin is denoted by the 2 digit International Organization for Standardization code; year isolated is denoted by 2 digits; and stage is denoted by "a" (acute, if Fiebig stage is unknown) or "1", "2", "3", "4", "5", or "6" (acute or early chronic, where the number or range corresponds to the Fiebig stage or range of stages when known).
**EPV = Env-pseudotyped virus

Supplemental Table 4. Mean neutralizing antibody titers (ID_{50}) by isolate and treatment group.

Isolate	Month	Mean (SD) neutralizing antibody titer (ID_{50})				
		Control (C1-4)	T1: N/N/NA/NA	T2: NA/NA/NA/NA	T3: D/D/NA/NA	T4: DA/DA/NA/NA
Tier 1 (Clade B)						
BaL.26	1.5	5 (0)	5 (0)	5 (0)	5 (0)	5 (0)
	3.5	5 (0)	5 (0)	5 (0)	5 (0)	5.3 (1.4)
	6.5	5 (0)	5 (0)	5 (0)	5 (0)	5.3 (1.4)
	12	5 (0)	5 (0)	5 (0)	5 (0)	5 (0)
MN.3	1.5	5 (0)	5 (0)	103.5 (125.4)	5 (0)	96.9 (78.6)
	3.5	5 (0)	6.1 (3.3)	323.7 (229.5)	8.5 (15.7)	424.1 (332.3)
	6.5	5 (0)	369.4 (329.5)	195 (130.8)	433 (248.8)	232.1 (150.6)
	12	5 (0)	17.9 (14.9)	14.1 (11.4)	34.5 (32.9)	21.5 (21.2)
SF162.LS	1.5	5 (0)	5 (0)	5 (0)	5 (0)	5 (0)
	3.5	5 (0)	5 (0)	8.4 (8)	10.7 (25.3)	14.8 (14)
	6.5	5 (0)	21.2 (13.5)	10.3 (12.3)	18.2 (15.4)	16.2 (15.7)
	12	5 (0)	5 (0)	5 (0)	5 (0)	5 (0)
Tier 1 (Clade C)						
MW965.26	1.5	5 (0)	5 (0)	5.7 (2.9)	5 (0)	7.3 (4.3)
	3.5	5 (0)	16.4 (14.1)	32.6 (24.1)	5 (0)	81.4 (144.1)
	6.5	5 (0)	66.2 (58.4)	46 (26.9)	51.8 (53.8)	100.1 (133)
	12	5 (0)	5.8 (2)	6.5 (3.3)	5.8 (2.3)	13.7 (16.3)
NP03.13	1.5	5 (0)	5 (0)	5 (0)	5 (0)	5 (0)
	3.5	5 (0)	5 (0)	5.4 (1.6)	5 (0)	6.4 (4.2)
	6.5	5 (0)	5 (0)	5 (0)	5.5 (2.2)	6.4 (4.3)
	12	5 (0)	5 (0)	5 (0)	5 (0)	5 (0)
TH023.6	1.5	5 (0)	5 (0)	6.9 (4.4)	5 (0)	8.3 (7.1)
	3.5	5 (0)	15.1 (14.3)	46.2 (44.6)	5.2 (1.1)	71.8 (84.9)
	6.5	5 (0)	54.9 (47.4)	65.7 (53.4)	77.8 (131.4)	109 (118.5)
	12	5 (0)	7.1 (4.7)	13 (11.4)	5.4 (1.5)	17.6 (13.3)
Tier 2 (Clade C)						
96ZM651.2	6.5	5 (0)	5 (0)	5 (0)	5 (0)	5 (0)

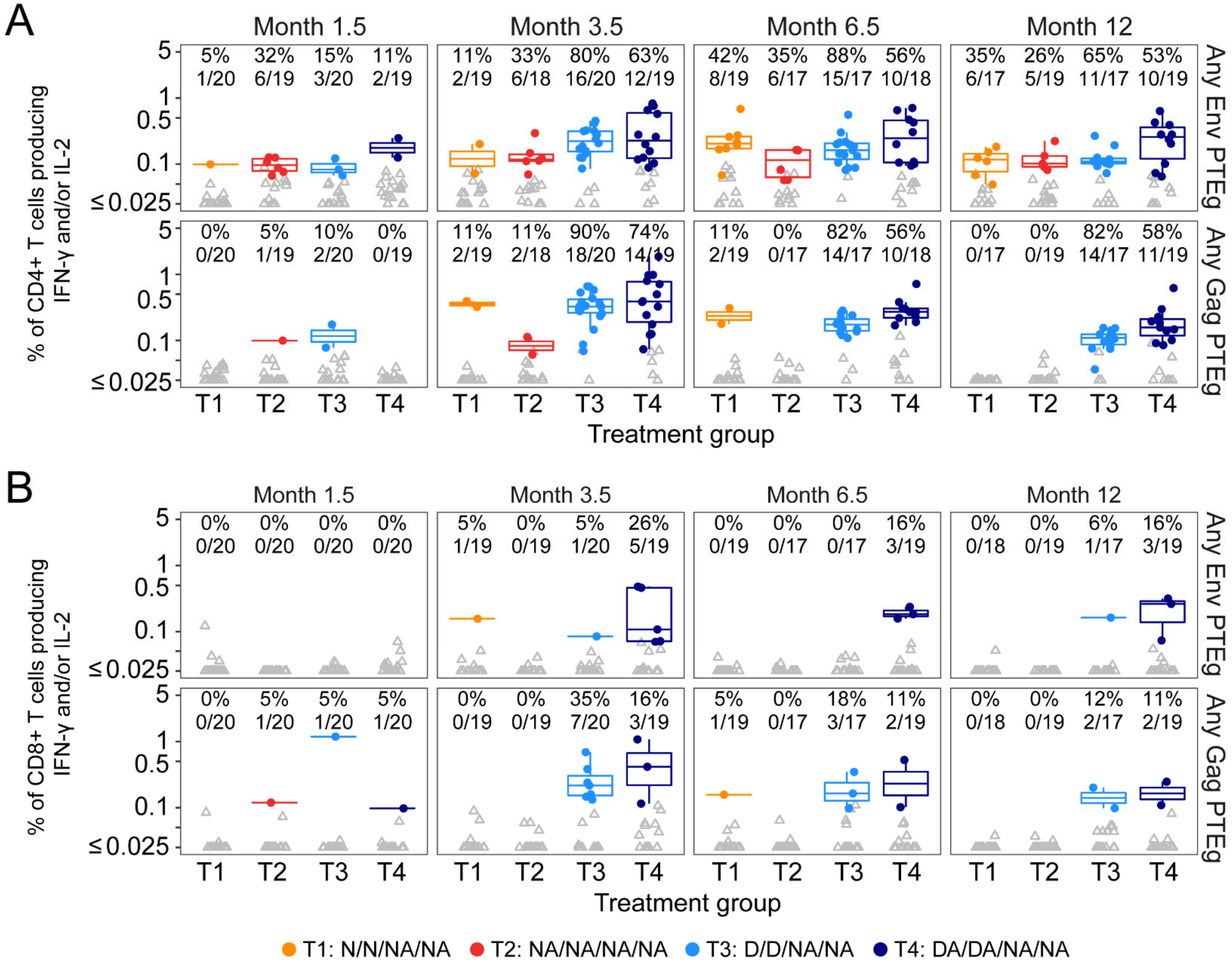

Supplemental Figure 1. CD4+ and CD8+ T-cell responses measured at different timepoints after vaccine administration. CD4+ and CD8+ T-cell responses were measured by ICS and expressed as the percentage of cells producing IL-2 and/or IFN-γ in the different treatment groups. (A) CD4+ and (B) CD8+ T-cell response rates and magnitudes among positive responders to Any Env or Gag PTE_g at 2 weeks post 2nd, 3rd, and 4th vaccinations and 6 months post 4th vaccination. The proportion of positive responders are shown at the top of each panel.

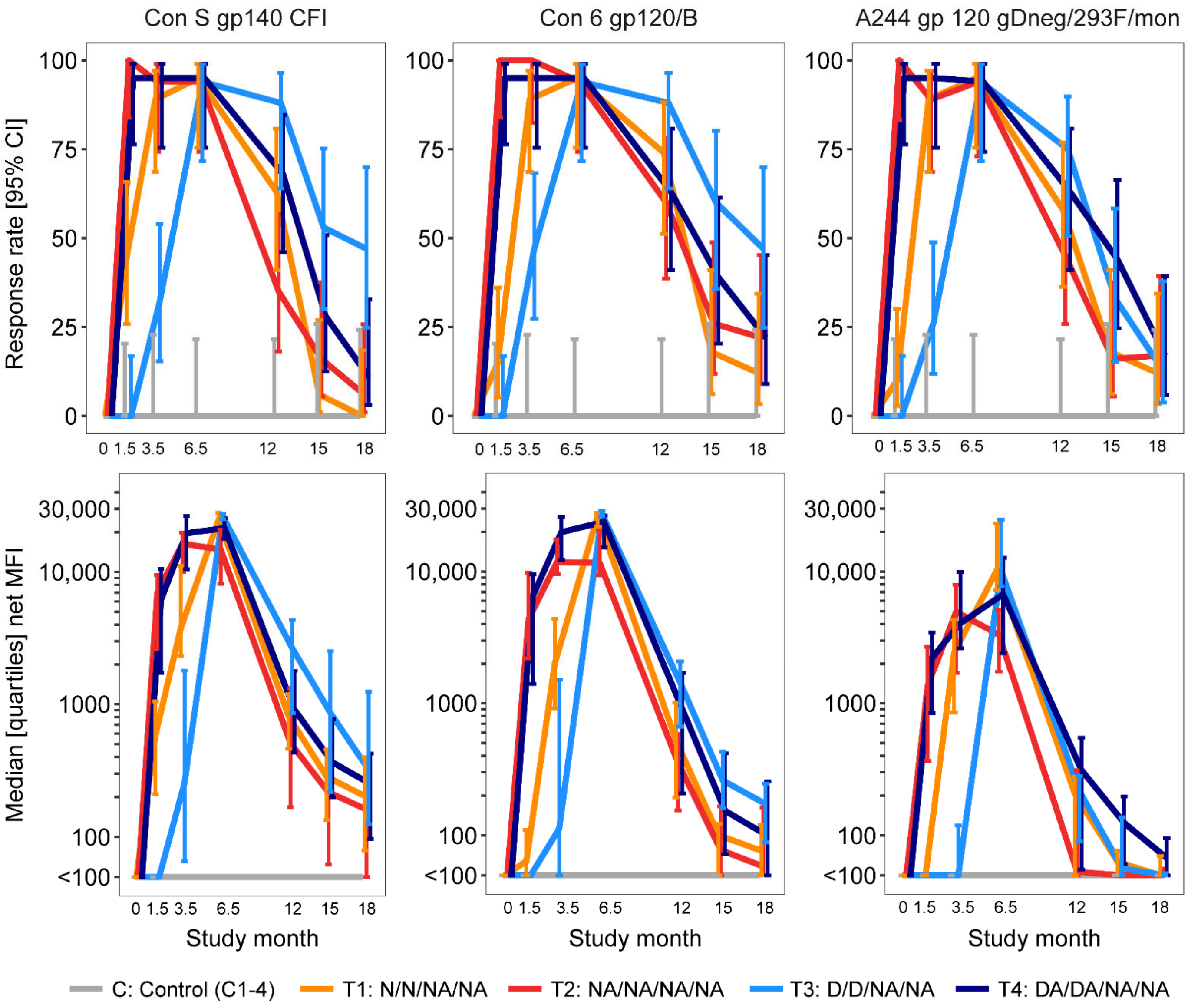

Supplemental Figure 2. Total IgG binding anti-Env antibody responses. IgG binding antibody response rates and magnitudes among positive responders were measured by BAMA at 2 weeks post 2nd, 3rd, and 4th vaccinations and the durability of the antibody response at 6, 9, and 12 months post 4th vaccination. IgG binding antibody to Con S gp140 CFI, Con 6 gp120/B and A244 gp120 gDneg/293F/mon are shown. The colored lines indicate the response rates and response magnitudes in the different treatment groups. Gray lines indicate the control (placebo) group.

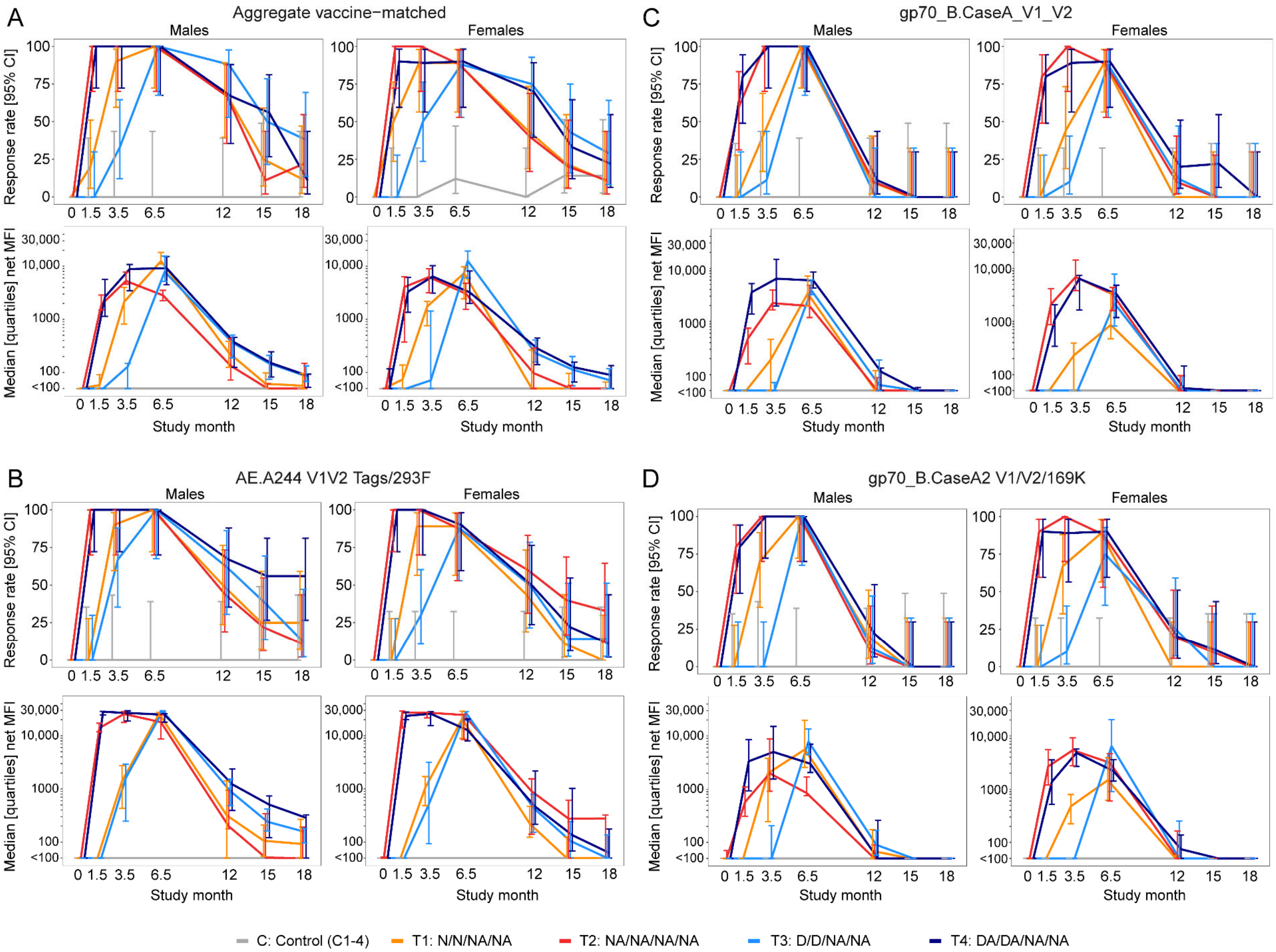
A
Aggregate vaccine−matched
Males
Females
Response rate [95% CI]
Median [quartiles] net MFI
Study month
B
AE.A244 V1V2 Tags/293F
C
gp70_B.CaseA_V1_V2
D
gp70_B.CaseA2 V1/V2/169K
C: Control (C1-4)
T1: N/N/NA/NA
T2: NA/NA/NA/NA
T3: D/D/NA/NA
T4: DA/DA/NA/NA

Supplemental Figure 3. Total IgG binding anti-Env antibody responses by sex. IgG binding antibody response rates and magnitudes among positive responders were measured by BAMA at 2 weeks post 2nd, 3rd, and 4th vaccinations and the durability of the antibody response at 6, 9, and 12 months post 4th vaccination. IgG binding antibody were measured against three vaccine matched antigens (aggregate-vaccine-matched responses), and three V1V2 antigens. The colored lines indicate the response rates and response magnitudes in the different treatment groups. Gray lines indicate the control (placebo) group. Responses to aggregate vaccine-matched antigens are considered as positive if positive response to any of the vaccine-matched antigens.

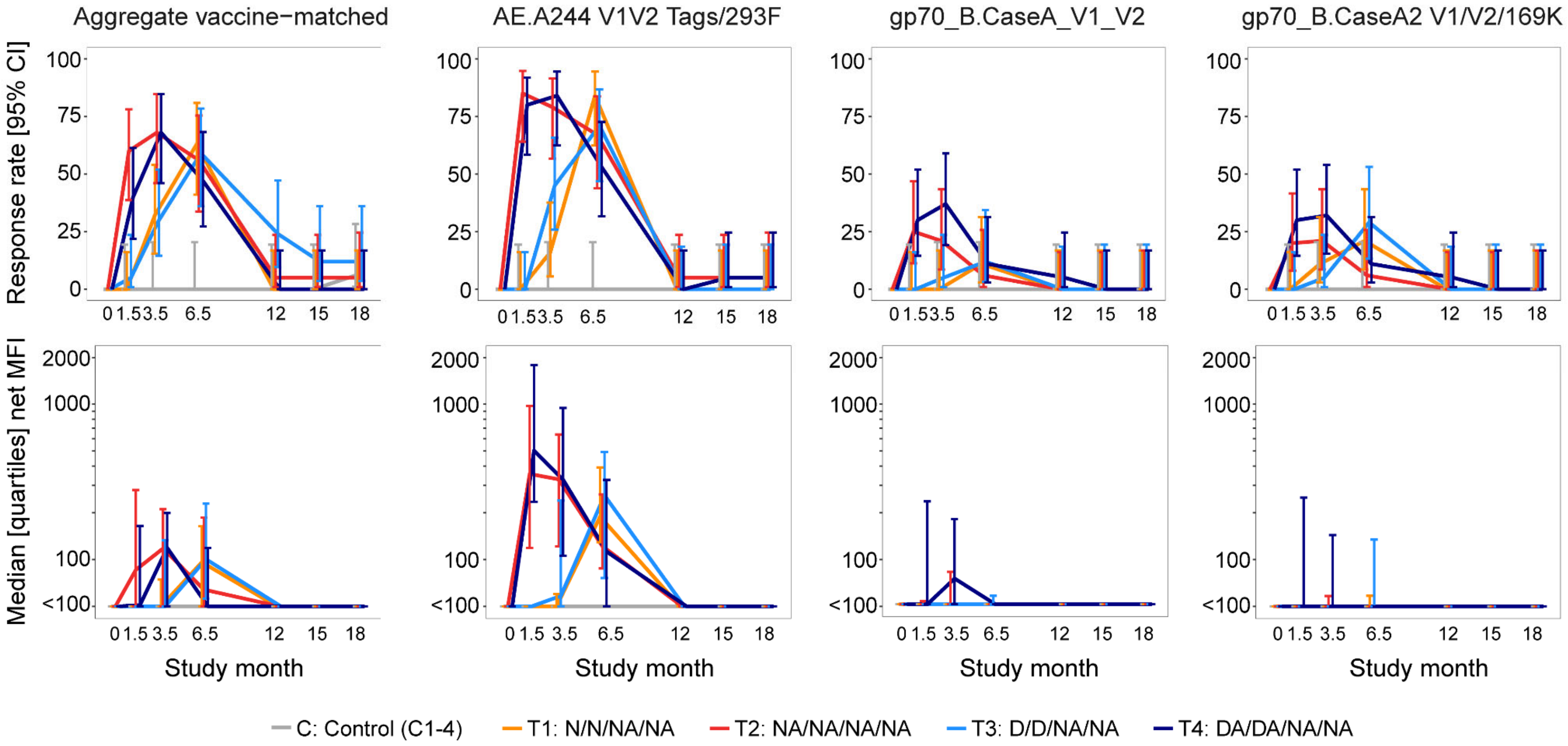

Supplemental Figure 4. Kinetics of IgG3 binding antibody responses. IgG3 antibody responses were measured by BAMA against aggregate vaccine-matched antigens and against three V1/V2 antigens. Responses were measured at 2 weeks post 2nd, 3rd, and 4th vaccinations and the durability responses at 6, 9, and 12 months post 4th vaccination. The colored lines indicate the response rates and response magnitudes among positive responders in the different treatment groups. Gray lines indicate the control (placebo) group. Responses to aggregate vaccine-matched antigens are considered as positive if positive response to any of the vaccine-matched antigens.

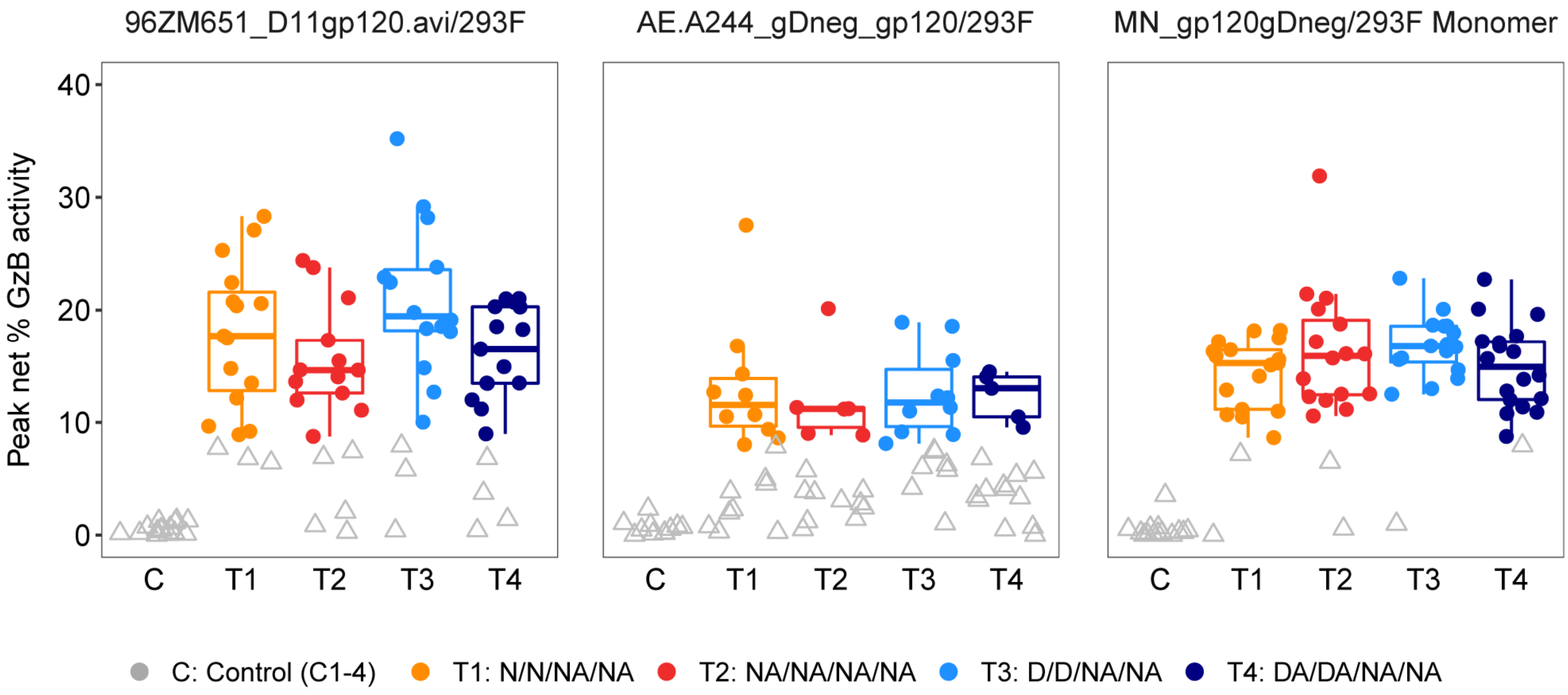

Supplemental Figure 5. Antibody-dependent cell mediated cytotoxicity (ADCC) peak net percent granzyme B activity at peak timepoint (month 6.5). Peak percentage of granzyme B activity at month 6.5 (2 weeks post 4th vaccination) for three different antigens in the different treatment groups are shown. Positive responses are shown in filled color circles while negative responses are shown in open grey triangles.

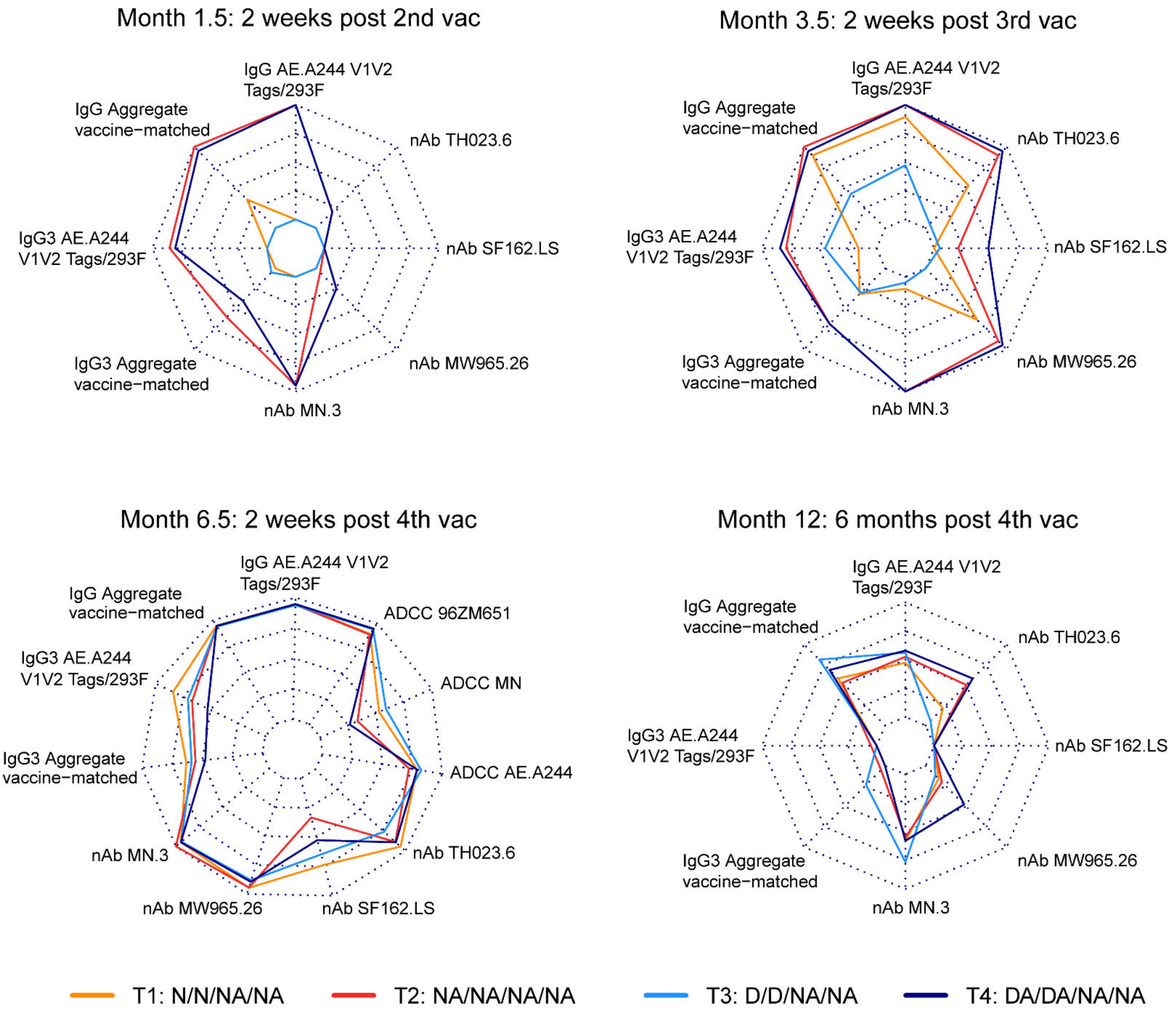

Supplemental Figure 6. Radar plots comparing response rates for a selected set of immune responses in the different treatment groups. Each spike represents the response rate for the immune response at the specified time point. The center of each plot is a response rate of 0%, and the circular lines indicate response rates of 25%, 50%, 75%, and 100%. Response rates at 2 weeks post 2nd, 3rd , 4th and 6 months post 4th vaccinations are shown. For ADCC responses, the comparisons of response rates between the four study groups were available only at Month 6.5.
